# Supplementary material for: Probabilistic edge weights fine-tune Boolean network dynamics
Source: PLoS Comput Biol. 2022 Oct 10;18(10):e1010536. doi: 10.1371/journal.pcbi.1010536 (PMC9584532; doi:10.1371/journal.pcbi.1010536)
Supplement: S1 Document — The document discusses cases of equivalence and cases where the two approaches are different, demonstrated through examples. (PDF) [file pcbi.1010536.s001.pdf]

S1 Document - Probabilistic Edge Weights Fine-tune Boolean Network Dynamics: Comparison between PEW and PBN

Dávid Deritei, Nina Kunšič, Péter Csermely

September 13, 2022

In this document we compare in detail the PBN framework and the PEW frameworks. We show a way to convert between Probabilistic Edge-Weights (PEW) into PNBs (Probabilistic Boolean Networks) in some special cases and discuss some of the differences.

1 Definitions

As a reminder, in PBNs one can specify a set of regulatory functions $F_i = \{f_1^i, f_2^i, ...f_n^i\}$ for each node i in the Boolean network. Each of these functions has an assigned probability within the set, $C_i = \{c_1^i, c_2^i, ...c_n^i\}$. The probabilities within each set add up to 1 ($\sum_j^n c_j^i = 1$). A *realisation* of the network is a single selected function for each node. The number of possible realizations is equal to the product of the node-function set sizes, i.e. $\Pi_i^N ||F_i||$. During every time-step a ξ random variable decides if the previous realisation is to be kept for time-step $t + 1$ or a new realization is to be selected. For a new realization the functions are re-drawn for each node i from their corresponding set F_i according to the probability distribution encoded in C_i.

In PEW models the function selection is conditioned on the value of the input node or clause. Moreover, the noise-function of the P_e operator in Equation (1) of the main manuscript can be any function that returns a binary value based on the weight parameters (e.g. Sigmoid, SDDS, etc.). This

is a key difference as compared to PBN-s and allows for a great degree of flexibility. This is demonstrated in the main manuscript via the reproduction of some of the SDDS models and the results of the Poret et. al. paper and further examples in the Application Note. For more details see the Appendices, Supplementary documents and the accompanying Jupyter notebooks. In the two applications described in the manuscript we demonstrate the PEW framework use the Bernoulli "coin-toss" as the noise function (draw from a binomial distribution). With this function it is indeed possible to convert PEW models into PBNs.
Here we demonstrate this conversion through an example. We would argue that in this case the PEW being a special case of the PBN framework does not necessarily reduce its value. On the contrary, models designed with specific edge-weights in mind (e.g. data driven models, where more is known about edge weights than about alternative node functions) can be converted into PBNs and further analyzed with the tools available for PBN models. On the other hand, if the PBN "view" is not necessary, the available tools provided along this manuscript and the classic BooleanNet framework is sufficient.

2 Conversion from PEW to PBN: Example of two nodes

We demonstrate the equivalence between PEW and in PBN simple example of two nodes, A and B. The simplest possible Boolean model would be the following:

$$A^* = B$$

The "truth table" representation of this model is:

In_B	f^A
0	0
1	1

We treat B as an input. Let's assume that the link from B to A is noisy. Thus we add the PEW operator $[p_1, p_2]$ with values $p_1 = 0.9$, $p_2 = 0.7$. The conditional probability table describing the effects of the operator is shown below.

B	P(A=0)	P(A=1)
0	0.3	0.7
1	0.1	0.9

In the next steps we show how this conditional probability table can be turned in to a set of PBN functions. First, we set the parameter $\xi = 1$. In this case in every time-step when A is updated we select from the f_i functions, according to probability distribution defined in C_i. In other words every time A is updated a new "realization" of the model is selected. The question is what are the functions and the corresponding probabilities given the above conditional probability table?

To determine this we first expand the columns of the original truth table so that the ratio of ones and zeros in each *row* (conditional on B) corresponds to the probabilities defined in the conditional probability table (i.e. the probabilities of the PEW operator). We assign uniform selection probabilities to each resulting column (function) where $c_i = 1/n$, where n is the number of functions. See below the expanded truth table for our working example:

B	f_1	f_2	f_3	f_4	f_5	f_6	f_7	f_8	f_9	f_{10}	
0	0	0	0	1	1	1	1	1	1	1	$\sum_i c_i * f_i(B=0) = 0.7$
1	0	1	1	1	1	1	1	1	1	1	$\sum_i c_i * f_i(B=1) = 0.9$
Ci	0.1	0.1	0.1	0.1	0.1	0.1	0.1	0.1	0.1	0.1	

Finally collapse the unique functions adding up their corresponding c_i probabilities. The final truth table of the PBN is shown below:

B	f_1	f_2	f_3	
0	0	0	1	$\sum_i c_i * f_i(B=0) = 0.7$
1	0	1	1	$\sum_i c_i * f_i(B=1) = 0.9$
Ci	0.1	0.2	0.7	

In Figure 2 we show that the PEW and the derived PBN are equivalent, as the transition probabilities are the same.

3 Conversion from PBN to PEW

It is theoretically possible to convert from PBN to PEW using the same formalism demonstrated in the example above. In the case of a single input

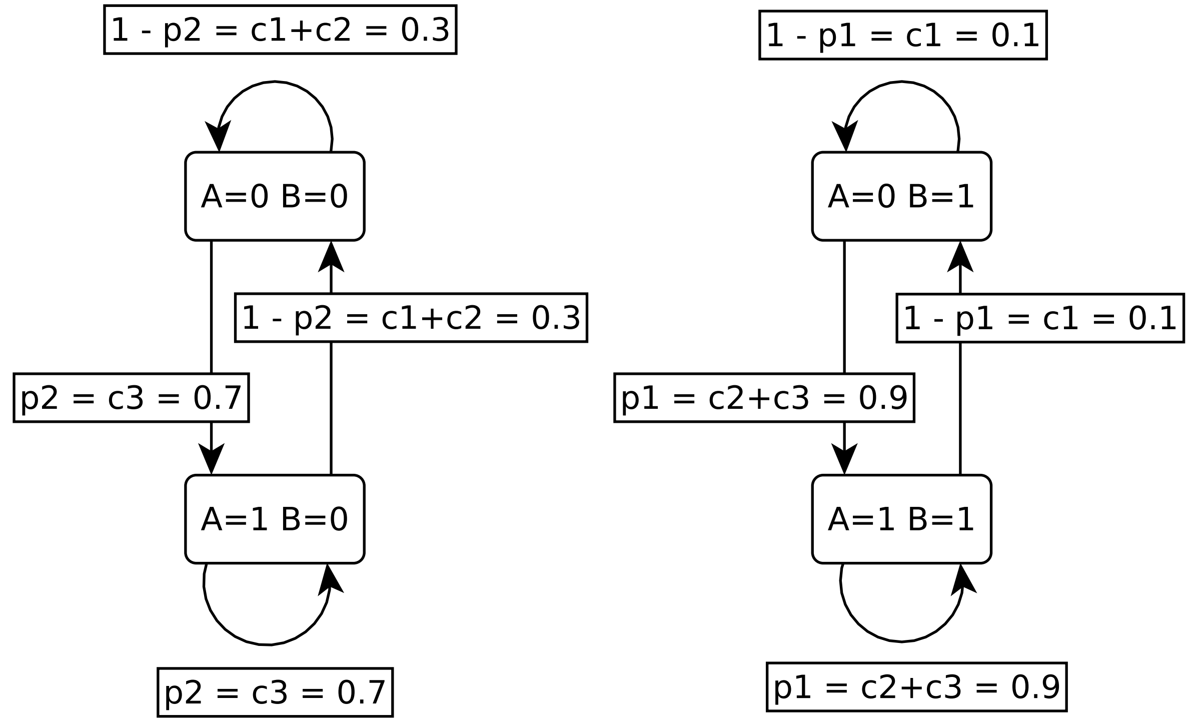

Figure 1: State transition graph of updating A with probabilities assigned to each edge. The edge descriptions include both the PEW probabilities and the PBN function probabilities. The labels p1 and p2 refer to the PEW operator probabilities and the labels c1,c2,c3 to the selection probabilities of the final PBN funtions.

for a node A from the node B, the weighted sum of the rows of the truth table (for the two values of B) determines the conditional

$$p_{on} = \sum_i c_i * f_i(B = 1)$$

and

$$p_{off} = \sum_i c_i * f_i(B = 0)$$

parameters respectively. This way the PBN function set of A can be transformed into a probabilistic edge weight from B to A.

In the case of multiple inputs all possible input combinations would need to be accounted for with a different PEW weight. For example if node A has two inputs B and C the PEW weights would be calculated as such:

$$p_{B=0,C=0} = \sum_i c_i * f_i(B = 0, C = 0)$$

$$p_{B=0,C=1} = \sum_i c_i * f_i(B = 0, C = 1)$$

$$p_{B=1,C=0} = \sum_i c_i * f_i(B = 1, C = 0)$$

$$p_{B=1,C=1} = \sum_i c_i * f_i(B = 1, C = 1)$$

This general conversion can become very complex as the number of input combinations grows exponentially with the number of inputs to a node. This means that generally one would need 2^k different PEW parameters for each PBN node with k number of inputs.

4 Final remarks

The above described general case (with any number of weight parameters) is currently not implemented in the code accompanying the manuscript but it can be a future upgrade. Moreover, in the general case one assumes that the subinputs are conditionally dependent, which is not necessarily the case. This means that the exponentially increasing number of parameters is a worst case scenario. In real biological systems many inputs are canalizing or simply ineffective, thus it is possible to come up with clever ways of reducing the number of necessary PEW parameters significantly. However this is beyond the scope of the current manuscript.
Furthermore the conversions described in this document are true only for the special case, where the PEW function f (from the general operator $P(f, w_{on}, w_{off})$) is the draw from a binomial distribution (which we also refer as the Bernoulli coin toss). The two main applications described in the manuscript both use this as the f function, yet in an additional application note we demonstrate some other use-cases where using a different f can generate behaviors that can be useful in other biological applications.
