# Supplementary material for: Probabilistic edge weights fine-tune Boolean network dynamics
Source: PLoS Comput Biol. 2022 Oct 10;18(10):e1010536. doi: 10.1371/journal.pcbi.1010536 (PMC9584532; doi:10.1371/journal.pcbi.1010536)
Supplement: S2 Document — (DOCX) [file pcbi.1010536.s002.docx]

# S2 Document - Probabilistic Edge Weights Fine-tune Boolean Network Dynamics

### Other Noisy Boolean models as special cases of PEW

**BNp with perturbation probability
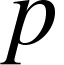
**

Reproducing perturbed Boolean Networks within the PEW framework is straightforward, by treating the full Boolean rule of a node as a hyper-edge and assigning
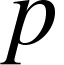
 and
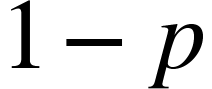
 Bernoulli noise for the OFF and ON states respectively. For a single node, this means applying the PEW operator on the full rule such as:


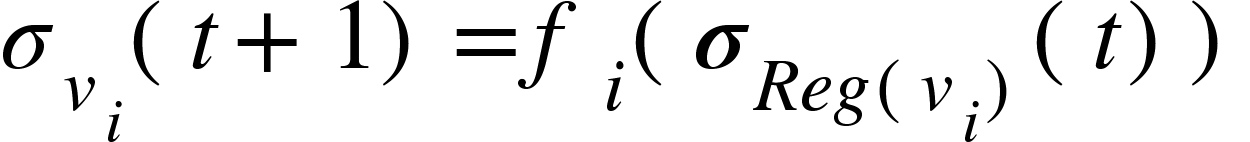
, becomes:


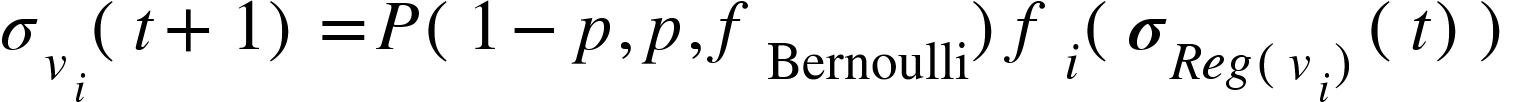
,

The PEW framework offers the added flexibility that one does not have to use the same
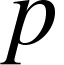
 probability of every node, in fact, all nodes can have a different noise distribution.

**SDDS with probabilities
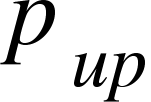
,
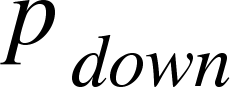
**


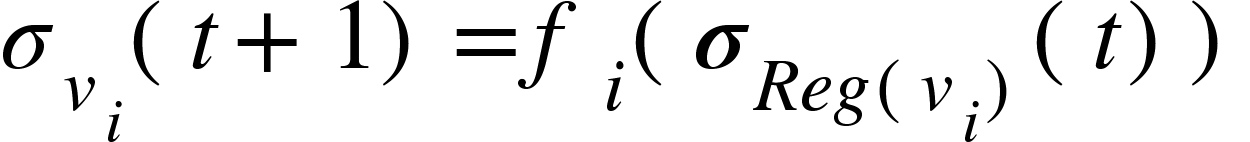
,


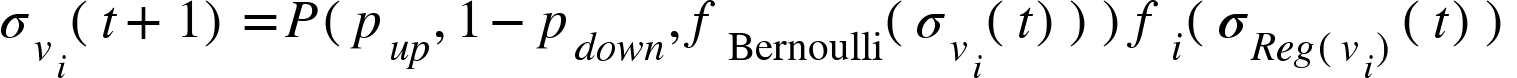
,

In this case
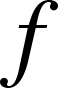
 also depends on the present value of the node
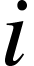
 and the conditional parameter is decided based on the comparison between
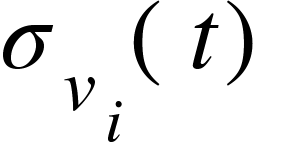
 and
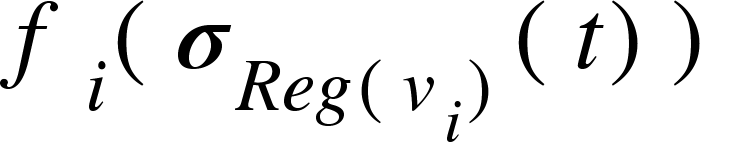


[For the reproduction of one of the results from Murrugarra et al.](https://www.codecogs.com/eqnedit.php?latex=f_i(%5Cboldsymbol%7B%5Csigma%7D_%7BReg(v_i)%7D(t))#0) [[19]](https://paperpile.com/c/9sbG9k/T8ahL) [see](https://www.codecogs.com/eqnedit.php?latex=f_i(%5Cboldsymbol%7B%5Csigma%7D_%7BReg(v_i)%7D(t))#0) the S7 Notebook ([Murrugarra_et_al_2012_paper_results.ipynb](https://github.com/deriteidavid/boolean2pew/blob/main/Murrugarra_et_al_2012_paper_results.ipynb))[.](https://www.codecogs.com/eqnedit.php?latex=f_i(%5Cboldsymbol%7B%5Csigma%7D_%7BReg(v_i)%7D(t))#0)

**PEWBNs are a special case of DBNs**

We would argue that the PEW method is a step toward the DBN framework in generality because the rules of some nodes with PEWs become stochastic and conditionally dependent on the value of the (subset of) parents. Nonetheless, it’s still more pragmatic to view PEW models as a separate framework because, as we show in the empirical applications, probabilistic edge weights are meant as very specific, targeted modifications to Boolean models and are easily made compatible with existing tools of Boolean network analysis. On the other hand, DBNs are likely the most general modeling framework of which PEWs models represent a special case.

**Comparison on PBNs and PEWBNs**

The relationship between **Probabilistic Boolean Networks** and PEW BNs is discussed in detail in S1 Document.
