# Supplementary material for: Probabilistic edge weights fine-tune Boolean network dynamics
Source: PLoS Comput Biol. 2022 Oct 10;18(10):e1010536. doi: 10.1371/journal.pcbi.1010536 (PMC9584532; doi:10.1371/journal.pcbi.1010536)
Supplement: S3 Document — Application Note: The document presents three more applications, where the PEW operators offer a relatively straightforward way to encode complex edge modulation, as well as examples for the usage of the general PEW operators, where the noise function is specified as well. (DOCX) [file pcbi.1010536.s003.docx]

# S3 Document: Probabilistic Edge Weights Fine-tune Boolean Network Dynamics

# Application Note

In this supplementary document to the manuscript entitled Probabilistic Edge Weights Fine-tune Boolean Network Dynamics by Deritei et al. we describe a few more simple applications to the PEW framework. The goal of these applications is to demonstrate the versatility of the method in different contexts. The note is accompanied by a Jupyter notebook titled Supplementary_Application_Note.ipynb/S3 Notebook where all the results can be reproduced.

## 1. Doubly Tyr-phosphorylated p27Kip1 partially reactivates Cdk4/CyclinD

In a 2012 study Ou et al. [1] described how the intrinsically disordered protein (IDP) p27Kip1 regulates the cell cycle entry at different levels of its phosphorylation. Unmodified p27 strongly inhibits the Cdk4/CyclinD complexes, thus also inhibiting cell cycle entry. Certain non-receptor tyrosine kinases (NRTK) can initiate the phosphorylation of p27. For example Abl (a NRTK) can phosphorylate p27 at Tyr88. This first modification does not change the behavior of p27 with regards to inhibition of Cdk4/CyclinD, however it opens p27 up in a way that allows for a second phosphorylation by another NRTK, Src. Src can phosphorylate p27 at Tyr 74. Intriguingly, the doubly modified p27 allows for an activation of Cdk4/CyclinD but only at a rate of ~20%, instead of full activation.

Here we propose a simple model that reproduces this phenomenon. The rules of the Boolean model are proposed as such:

Cdk4*= not p27 and not p27_tyr88 and **[1,0.2]** not p27_tyr88_tyr74

p27 *= NRTK

p27_tyr88 *= p27 and (Abl or Src)

p27_tyr88_tyr74 *= p27_tyr88_tyr74 or (p27_tyr88 and Src)

The Boolean rules follow the biological description above, with a highlighted PEW operator in the rule of Cdk4. Cdk4 is inhibited by all versions of p27, with the exception of the doubly phosphorylated p27, represented in the model by the variable p27_tyr88_tyr74, which allows for a 20% activation of Cdk4. Here we encode this explicitly with a PEW operator on the inhibitory edge from p27_tyr88_tyr74 to Cdk4.

On Figure 1 we show an ensemble simulation of the above model. Certain NRTK phosphorylate p27, resulting in its binding and nuclear translocation of Cylcin D1/CDK4 complex [4]. However when p27 is not phosphorylated on Tyr88 and Tyr74 it strongly inhibits CDK4 activation. In our simulation initially Cdk4 is ON, however as we turn on the NRTK signaling p27 binding immediately inhibits Cdk4. Next we sequentially turn on Abl and Src, allowing for the activation of the nodes representing the phosphorylated varaints of p27. It is indeed when only p27_tyr88_tyr74 is active that Cdk4 reactivates and stabilizes at 20% due to the noisy operator on the edge. Reproducing the same behavior with other methods would be much less straightforward.


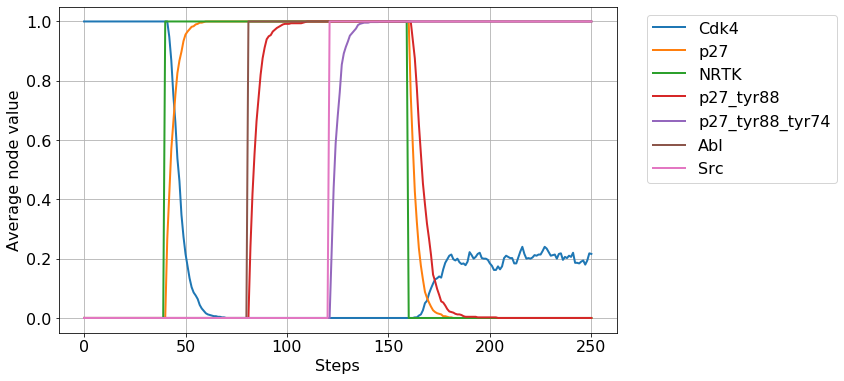


*Figure 1: Ensemble simulation of the p27 model proposed above. The input nodes NRTK, Abl and Src are turned on sequentially. Cdk4 remains off until the first two p27 variations are on, but goes to a 20% activation as soon as p27_tyr88_tyr74 becomes the dominant p27 version and the others are degraded. The simulation is done with 500 independently simulated cells using general asynchronous update.*

It is important to note that we made p27_tyr88_tyr74 self-sustaining by adding a self-loop in its rule. These self-loops are useful in cases where the degradation of a molecule happens at a slower rate than its activation (or none at all). However, often these self-loops are not biologically plausible. In the SDDS framework of Murrugarra et al. [2] this is addressed by using a different degradation propensity than the activation propensity. In the next application we will demonstrate the flexibility of the general PEW framework by using the SDDS function in a PEW operator, instead of the default Bernoulli coin-toss.

## 2. E2F hysteresis is reproduced by PEW operator combined with SDDS function

In one of the classic studies of the restriction point passage Yao et al. [3] show that with a sufficiently increased serum pulse the E2F “memorizes” and maintains its ON state independently of continuous serum stimulation. This is a classic case of self-sustaining feedback loop mechanisms which is well understood through the modeling framework of stable motifs (discussed in more detail in the main manuscript). However it is often the case that the self-sustaining feedback loops in Boolean networks are created artificially (by drawing artificial edges or self-loops), so that the hysteresis observed in the experiments is reproduced in the emergent dynamics of the Boolean model.

Here we present an alternative, where no artificial edge is necessary. We use the functional formalism of the stochastic discrete dynamical systems (SDDS) proposed by Murrugarra et al [2] combined with the PEW framework. Namely we use a PEW operator on the rule of the E2F where we use the SDDS function instead of the default Bernoulli coin-toss and use a very small degradation propensity, so that the activation is quick but its degradation is very slow. The rules of the model are the following:

CyclinD*= (Myc and GF)

CyclinE*= E2F

E2F*= **[SDDS,1,0.99]** ((not RB) and Myc)

Myc*= GF

RB*= (not CyclinD) and (not CyclinE)

In defining the rules of the Boolean model we use the interaction scheme offered by Yao et al. in their paper, with the exception of the E2F self-loop. The self-sustaining behavior is now ensured by the PEW operator using the SDDS formalism as its noise function.

*(A technical note: any noise function can be specified in the accompanying Python file PEW_functions.py. One has only to define the function and use its name in the PEW operator in the Boolean rule, as it’s done above, the software can parse this general case as well. Different PEW operators with different noise functions can be mixed in the same model.)*

In this case the SDDS noise function ensures that when E2F is upregulated the regulation is successful with probability p=1. However when it’s downregulated it is only successful with probability p=1-0.99=0.01. This means that when E2F1 is turned on it’s very difficult to turn it off, but it still has a slow degradation. The same effect can be reached with a noisy self-loop, which is applied in the EMT model presented in the main manuscript.

On Figure 2 we show the ensemble simulation of the model with and without an initial pulse.


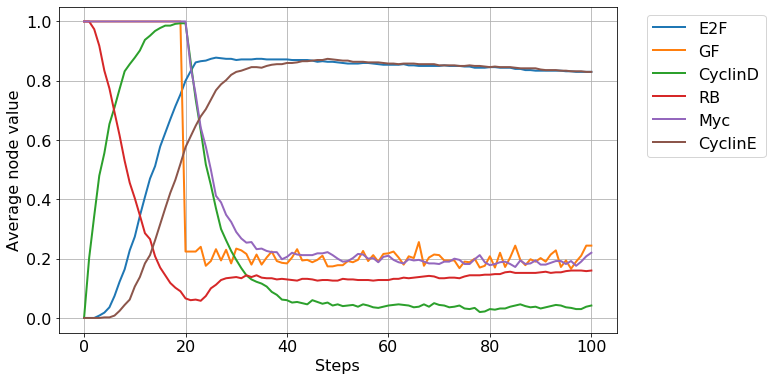


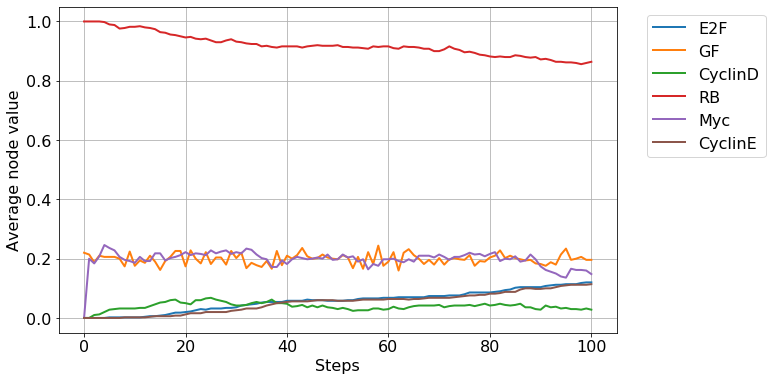


*Figure 2: Top - an initial growth factor pulse raises the E2F and RB levels, but after the removal of the pulse E2F and CyclinE both remain high with a slight decline. Bottom - the default 20% serum concentration is not sufficient to “kickstart” the E2F hysteresis, thus all molecules remain on a pre-restriction point passage level. The simulation is done with 500 independently simulated cells using general asynchronous update.*

##

## 3. Exponential noise function produces sophisticated node behaviors

Here we expand the noise function idea a little bit further and examine what happens if the success of an activation and/or degradation is determined by drawing from an exponential distribution (instead of a binomial distribution).

We define the following exponential distribution:

[
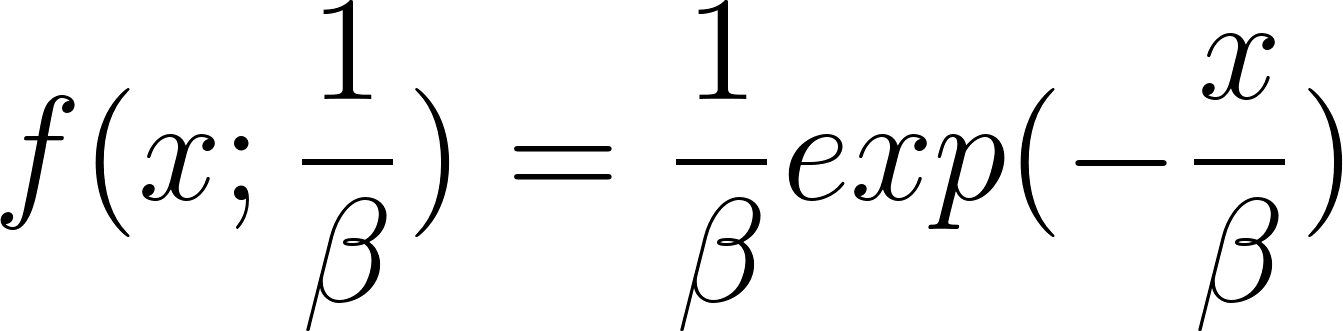
](https://www.codecogs.com/eqnedit.php?latex=f(x%3B%5Cfrac%7B1%7D%7B%5Cbeta%7D)%20%3D%20%5Cfrac%7B1%7D%7B%5Cbeta%7Dexp(-%5Cfrac%7Bx%7D%7B%5Cbeta%7D)%20#0),

for [
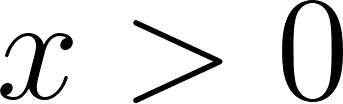
](https://www.codecogs.com/eqnedit.php?latex=x%3E0#0) and 0 elsewhere. [
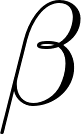
](https://www.codecogs.com/eqnedit.php?latex=%5Cbeta#0) is the scale parameter of the distribution. For further details on the implementation see the [numpy documentation](https://numpy.org/doc/stable/reference/random/generated/numpy.random.exponential.html) of the exponential distribution.

In a PEW operator one can specify a different scale parameter for downregulation or upregulation (w_up, w_down). Biologically this would correspond to a case where the success of two molecules interacting follows some exponential trend. The PEW operator can be written as:

[
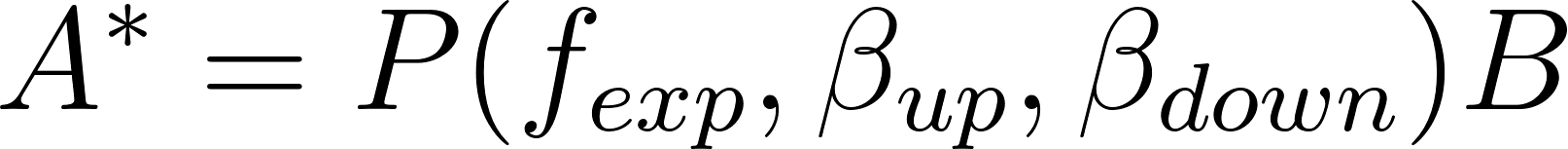
](https://www.codecogs.com/eqnedit.php?latex=A%5E*%3D%20P(f_%7Bexp%7D%2C%20%5Cbeta_%7Bup%7D%2C%20%5Cbeta_%7Bdown%7D)%20B#0)

Where [
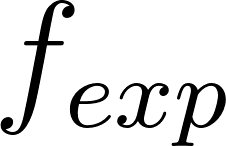
](https://www.codecogs.com/eqnedit.php?latex=f_%7Bexp%7D#0) returns 1 if the value [
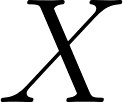
](https://www.codecogs.com/eqnedit.php?latex=X#0) drawn from the [
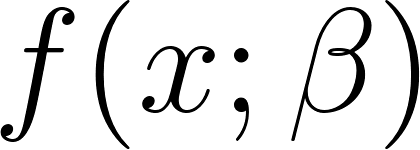
](https://www.codecogs.com/eqnedit.php?latex=f(x%3B%5Cbeta)#0) distribution is [
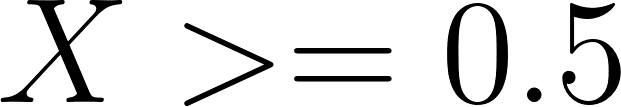
](https://www.codecogs.com/eqnedit.php?latex=X%20%3E%3D%200.5#0) and returns 0 if is [
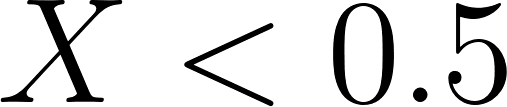
](https://www.codecogs.com/eqnedit.php?latex=X%3C0.5#0). The value of [
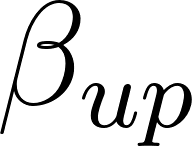
](https://www.codecogs.com/eqnedit.php?latex=%5Cbeta_%7Bup%7D#0) is used to generate the distribution if [
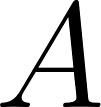
](https://www.codecogs.com/eqnedit.php?latex=A#0) is upregulated [
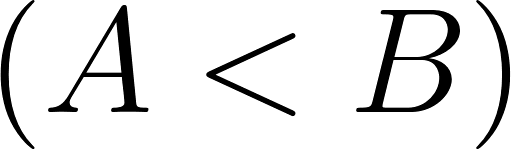
](https://www.codecogs.com/eqnedit.php?latex=(A%3CB)#0) and [
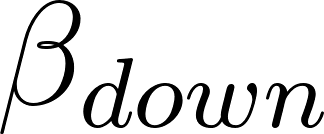
](https://www.codecogs.com/eqnedit.php?latex=%5Cbeta_%7Bdown%7D#0) is used if A is being downregulated [
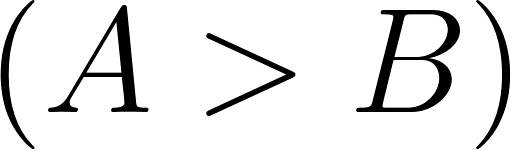
](https://www.codecogs.com/eqnedit.php?latex=(A%3EB)#0). If [
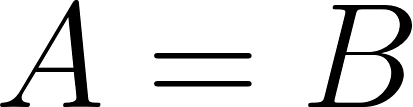
](https://www.codecogs.com/eqnedit.php?latex=A%3DB#0) than $f_{exp}$ returns their present value (i.e. nothing changes).

Figure 3 shows the behavior of this operator on a simple model with different [
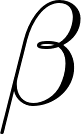
](https://www.codecogs.com/eqnedit.php?latex=%5Cbeta#0) parameters on the following model (in this cases [
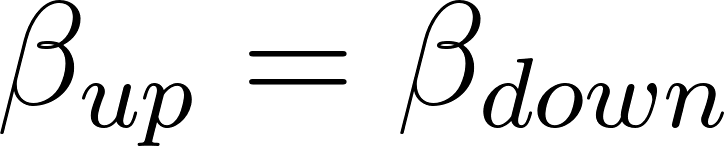
](https://www.codecogs.com/eqnedit.php?latex=%5Cbeta_%7Bup%7D%20%3D%20%5Cbeta_%7Bdown%7D#0)):

A*=[f_exp,0.1,0.1] B

C*=[f_exp,0.15,0.15] B

D*=[f_exp,0.2,0.2] B

E*=[f_exp,0.25,0.25] B


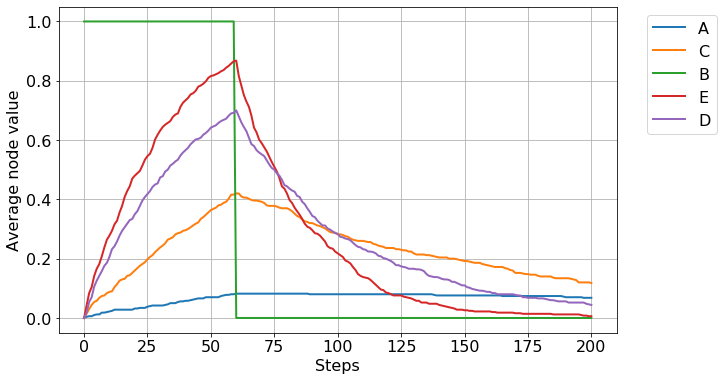


*Figure 3. The behavior of nodes reacting with the exponential noise function. The* [*
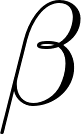
*](https://www.codecogs.com/eqnedit.php?latex=%5Cbeta#0) *values are 0.1, 0.15, 0.2, 0.25 respectively. The simulation is done with 500 independently simulated cells using general asynchronous update.*

One can interpret this function as a sort of tunable “reactivity”, from quickly reacting nodes to more slowly reacting ones. With different [
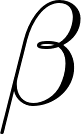
](https://www.codecogs.com/eqnedit.php?latex=%5Cbeta#0) parameters for degradation and for activation different asymmetries in reactivity can be implemented.

The transition rates and shapes are akin to ones resulting the Michaelis-Menten kinetics and behaviors possible only in other ODE models [5].

# References

[1] Ou, L., Waddell, M. B., & Kriwacki, R. W. (2012). Mechanism of cell cycle entry mediated by the intrinsically disordered protein p27Kip1. *ACS chemical biology*, *7*(4), 678-682.

[2] Murrugarra, D., Veliz-Cuba, A., Aguilar, B., Arat, S., & Laubenbacher, R. (2012). Modeling stochasticity and variability in gene regulatory networks. EURASIP Journal on Bioinformatics and Systems Biology, 2012(1), 1-11.

[3] Yao, G., Lee, T. J., Mori, S., Nevins, J. R., & You, L. (2008). A bistable Rb–E2F switch underlies the restriction point. *Nature cell biology*, *10*(4), 476-482.

[4] Ciarallo, S., Subramaniam, V., Hung, W., Lee, J. H., Kotchetkov, R., Sandhu, C., ... & Slingerland, J. M. (2002). Altered p27Kip1 phosphorylation, localization, and function in human epithelial cells resistant to transforming growth factor β-mediated G1 arrest. *Molecular and cellular biology*, *22*(9), 2993-3002.

[5] Kim, J. K., & Tyson, J. J. (2020). Misuse of the Michaelis–Menten rate law for protein interaction networks and its remedy. *PLoS Computational Biology*, *16*(10), e1008258.
