# Supplementary material for: Probabilistic edge weights fine-tune Boolean network dynamics
Source: PLoS Comput Biol. 2022 Oct 10;18(10):e1010536. doi: 10.1371/journal.pcbi.1010536 (PMC9584532; doi:10.1371/journal.pcbi.1010536)
Supplement: S4 Document — (DOCX) [file pcbi.1010536.s004.docx]

# S4 Document - Probabilistic Edge Weights Fine-tune Boolean Network Dynamics

### Boolean rules with and without PEW operators

**Application 1 - the simplified EMT model:**

*SNAI1 positive feedback loop* - SNAI1 inhibits transcription of miR-34a [[1]](https://paperpile.com/c/xuXymr/2IBX), which in turn inhibits SNAI1 translation resulting in decreased SNAI1 mRNA and protein levels [[2,3]](https://paperpile.com/c/xuXymr/RPoWA+eqLn9). Additionally, SNAI1 also inhibits HNF4A transcription [[4]](https://paperpile.com/c/xuXymr/6szzv), which positively regulates miR-122 transcription [[5]](https://paperpile.com/c/xuXymr/6kC7A), and miR-122, in turn, inhibits SNAI1 translation [[6]](https://paperpile.com/c/xuXymr/QfP1P).

*TGFB - miR200 inhibition* - Non-canonical TGFB pathway results in PI3K/AKT pathway activation [[7]](https://paperpile.com/c/xuXymr/QjE5t), leading to decreased GSK3B nuclear localization [[7,8]](https://paperpile.com/c/xuXymr/QjE5t+mevbV)] nuclear GSK3B-mediated DNMT1 phosphorylation and degradation [[9,10]](https://paperpile.com/c/xuXymr/vVDmp+5pwYg), thus decreasing miR-200 promoter methylation [[11]](https://paperpile.com/c/xuXymr/46uaD).

The remaining interactions in the simplified model are all canonical and supported by multiple papers referenced in the main text, such as [[12–15]](https://paperpile.com/c/xuXymr/zIAnT+I56GJ+GN1Zz+nKoHa).

**Wild type model:**

TGFB *= TGFB

SMAD *= TGFB or ZEB1

ZEB1 *= SMAD and not miR200

minter1 *= TGFB

minter2 *= minter1

miR200 *= **[1,0.05]**(not ZEB1) and not SNAI1 and not minter2

inter1 *= TGFB

inter2 *= inter1

inter3 *= inter2

inter4 *= inter3

inter5 *= inter4

inter6 *= inter5 or **[0.9,0]**SNAI1

SNAI1 *= inter6

Ecadherin *= not SNAI1 and not ZEB1

**Mutant model** (the only difference to compared to the wild type is in the PEW parameters)**:**

TGFB *= TGFB

SMAD *= TGFB or ZEB1

ZEB1 *= SMAD and not miR200

minter1 *= TGFB

minter2 *= minter1

miR200 *= **[1,0.99]**(not ZEB1) and not SNAI1 and not minter2

inter1 *= TGFB

inter2 *= inter1

inter3 *= inter2

inter4 *= inter3

inter5 *= inter4

inter6 *= inter5 or **[0.9,0]** SNAI1

SNAI1 *= inter6

Ecadherin *= not SNAI1 and not ZEB1

**Application 2 - Sizek et al. model (for all Boolean rules and citations see** [**SI Table 1**](https://storage.googleapis.com/plos-corpus-prod/10.1371/journal.pcbi.1006402/2/pcbi.1006402.s015.pdf?X-Goog-Algorithm=GOOG4-RSA-SHA256&X-Goog-Credential=wombat-sa%40plos-prod.iam.gserviceaccount.com%2F20211208%2Fauto%2Fstorage%2Fgoog4_request&X-Goog-Date=20211208T114449Z&X-Goog-Expires=86400&X-Goog-SignedHeaders=host&X-Goog-Signature=5b92077a5b3f141aca14dbb569c0ab3cd9791f9b465870c79118b7537e7976b662f2daf50952bd1f99cc6e39bc057f2ef1176475f4dfda3c74f72c349ce2f819eec02843c2ae4ce463b93256e899c3e913694e4da1e340ad537bfa42fbf79aa8c758b5a2059c1fd71a49097fc51fa71e8f63cf811dfcdf60a44b8784dcb6baaa5ce92d2bc360f5642a6964898958b2307e4115f301dce0d6b9d5925b7d94c83d353a673636ddd3e9e2417873c3e32f8f2284e22bf2582ac12a5ae11568d426c542f2b86200590331308733c20575cb53dc5b1949337bdf71efeaffab85a9ade000ff6d6d2c1ae649665d61a108889b754ef615dca0dfd2ffdcd3aefbb5396817) **of** [[16]](https://paperpile.com/c/xuXymr/OG3q8)**)**

Relevant interactions are highlighted in bold.

MCL_1 *= not Casp3 and not Casp2 and (not GSK3 or (AKT_B and (ERK or not E2F1) ) ) and **(not ((Cdk1 and CyclinB)** and U_Kinetochores) )

BCLXL *= not Casp3 and (BCL2 or not BAD) and ( not U_Kinetochores or ( Plk1 and **(not (CyclinB and Cdk1)** or (BCL2 and MCL_1) ) ) or ( (BCL2 and MCL_1) and **not (CyclinB and Cdk1)** ) )

BCL2 *= not (Casp3 or BAD or BIM or BIK) and ( not U_Kinetochores or (MCL_1 and BCLXL) or (Plk1 and (BCLXL or MIAPsCL_1 or **not (Cdk1 and CyclinB)** ) ) )

**With PEW operators:**

MCL_1 *= not Casp3 and not Casp2 and (not GSK3 or (AKT_B and (ERK or not E2F1) ) ) and **(not ([0.5,0](Cdk1 and CyclinB)** and U_Kinetochores) )

BCLXL *= not Casp3 and (BCL2 or not BAD) and ( not U_Kinetochores or ( Plk1 and **(not [0.5,0] (CyclinB and Cdk1)** or (BCL2 and MCL_1) ) ) or ( (BCL2 and MCL_1) and **not (CyclinB and Cdk1)** ) )

BCL2 *= not (Casp3 or BAD or BIM or BIK) and ( not U_Kinetochores or (MCL_1 and BCLXL) or (Plk1 and (BCLXL or MIAPsCL_1 or **not [0.5,0] (Cdk1 and CyclinB)** ) ) )

1. [Siemens H, Jackstadt R, Hünten S, Kaller M, Menssen A, Götz U, et al. miR-34 and SNAIL form a double-negative feedback loop to regulate epithelial-mesenchymal transitions. Cell Cycle. 2011;10: 4256–4271.](http://paperpile.com/b/xuXymr/2IBX)

2. [Kim NH, Kim HS, Li X-Y, Lee I, Choi H-S, Kang SE, et al. A p53/miRNA-34 axis regulates Snail1-dependent cancer cell epithelial-mesenchymal transition. J Cell Biol. 2011;195: 417–433.](http://paperpile.com/b/xuXymr/RPoWA)

3. [Yan X, Zhang D, Wu W, Wu S, Qian J, Hao Y, et al. Mesenchymal Stem Cells Promote Hepatocarcinogenesis via lncRNA-MUF Interaction with ANXA2 and miR-34a. Cancer Res. 2017;77: 6704–6716.](http://paperpile.com/b/xuXymr/eqLn9)

4. [Yang M, Li S-N, Anjum KM, Gui L-X, Zhu S-S, Liu J, et al. A double-negative feedback loop between Wnt-β-catenin signaling and HNF4α regulates epithelial-mesenchymal transition in hepatocellular carcinoma. J Cell Sci. 2013;126: 5692–5703.](http://paperpile.com/b/xuXymr/6szzv)

5. [Li Z-Y, Xi Y, Zhu W-N, Zeng C, Zhang Z-Q, Guo Z-C, et al. Positive regulation of hepatic miR-122 expression by HNF4α. J Hepatol. 2011;55: 602–611.](http://paperpile.com/b/xuXymr/6kC7A)

6. [Jin Y, Wang J, Han J, Luo D, Sun Z. MiR-122 inhibits epithelial-mesenchymal transition in hepatocellular carcinoma by targeting Snail1 and Snail2 and suppressing WNT/β-cadherin signaling pathway. Exp Cell Res. 2017;360: 210–217.](http://paperpile.com/b/xuXymr/QfP1P)

7. [Hamidi A, Song J, Thakur N, Itoh S, Marcusson A, Bergh A, et al. TGF-β promotes PI3K-AKT signaling and prostate cancer cell migration through the TRAF6-mediated ubiquitylation of p85α. Sci Signal. 2017;10. doi:](http://paperpile.com/b/xuXymr/QjE5t)[10.1126/scisignal.aal4186](http://dx.doi.org/10.1126/scisignal.aal4186)

8. [Bautista SJ, Boras I, Vissa A, Mecica N, Yip CM, Kim PK, et al. mTOR complex 1 controls the nuclear localization and function of glycogen synthase kinase 3β. J Biol Chem. 2018;293: 14723–14739.](http://paperpile.com/b/xuXymr/mevbV)

9. [Lin R-K, Hsieh Y-S, Lin P, Hsu H-S, Chen C-Y, Tang Y-A, et al. The tobacco-specific carcinogen NNK induces DNA methyltransferase 1 accumulation and tumor suppressor gene hypermethylation in mice and lung cancer patients. J Clin Invest. 2010;120: 521–532.](http://paperpile.com/b/xuXymr/vVDmp)

10. [Fang Q-L, Yin Y-R, Xie C-R, Zhang S, Zhao W-X, Pan C, et al. Mechanistic and biological significance of DNA methyltransferase 1 upregulated by growth factors in human hepatocellular carcinoma. Int J Oncol. 2015;46: 782–790.](http://paperpile.com/b/xuXymr/5pwYg)

11. [Sui C-J, Zhou Y-M, Shen W-F, Dai B-H, Lu J-J, Zhang M-F, et al. Long noncoding RNA GIHCG promotes hepatocellular carcinoma progression through epigenetically regulating miR-200b/a/429. J Mol Med . 2016;94: 1281–1296.](http://paperpile.com/b/xuXymr/46uaD)

12. [Steinway SN, Zañudo JGT, Ding W, Rountree CB, Feith DJ, Loughran TP Jr, et al. Network modeling of TGFβ signaling in hepatocellular carcinoma epithelial-to-mesenchymal transition reveals joint sonic hedgehog and Wnt pathway activation. Cancer Res. 2014;74: 5963–5977.](http://paperpile.com/b/xuXymr/zIAnT)

13. [Celià-Terrassa T, Bastian C, Liu DD, Ell B, Aiello NM, Wei Y, et al. Hysteresis control of epithelial-mesenchymal transition dynamics conveys a distinct program with enhanced metastatic ability. Nature Communications. 2018. doi:](http://paperpile.com/b/xuXymr/I56GJ)[10.1038/s41467-018-07538-7](http://dx.doi.org/10.1038/s41467-018-07538-7)

14. [Gregory PA, Bracken CP, Smith E, Bert AG, Wright JA, Roslan S, et al. An autocrine TGF-beta/ZEB/miR-200 signaling network regulates establishment and maintenance of epithelial-mesenchymal transition. Mol Biol Cell. 2011;22: 1686–1698.](http://paperpile.com/b/xuXymr/GN1Zz)

15. [Gregory PA, Bert AG, Paterson EL, Barry SC, Tsykin A, Farshid G, et al. The miR-200 family and miR-205 regulate epithelial to mesenchymal transition by targeting ZEB1 and SIP1. Nat Cell Biol. 2008;10: 593–601.](http://paperpile.com/b/xuXymr/nKoHa)

16. [Sizek H, Hamel A, Deritei D, Campbell S, Ravasz Regan E. Boolean model of growth signaling, cell cycle and apoptosis predicts the molecular mechanism of aberrant cell cycle progression driven by hyperactive PI3K. PLoS Comput Biol. 2019;15: e1006402.](http://paperpile.com/b/xuXymr/OG3q8)
