# Supplementary material for: Probabilistic edge weights fine-tune Boolean network dynamics
Source: PLoS Comput Biol. 2022 Oct 10;18(10):e1010536. doi: 10.1371/journal.pcbi.1010536 (PMC9584532; doi:10.1371/journal.pcbi.1010536)
Supplement: S1 Notebook — (HTML) [file pcbi.1010536.s005.html]

Murrugarra\_et\_al\_2012\_paper\_results


In [1]:

```
import random
import boolean2pew as b2p
import pandas as pd
import numpy as np
import EnsembleSimulation as es
```

In [2]:

```
def general_async_pick( lines ):
    line = [ random.choice( lines )]
    return line

def merge_two_dicts(x, y):
    z = x.copy()   # start with x's keys and values
    z.update(y)    # modifies z with y's keys and values & returns None
    return z
```

## Reproducing results of the Murrugarra et al. paper¶

Murrugarra, D., Veliz-Cuba, A., Aguilar, B., Arat, S., & Laubenbacher, R. (2012). Modeling stochasticity and variability in gene regulatory networks. EURASIP Journal on Bioinformatics and Systems Biology, 2012(1), 1-11.   
https://bsb-eurasipjournals.springeropen.com/articles/10.1186/1687-4153-2012-5

For this method a slight modification in the rule parser is needed. Since in the case of SDDS-s the noise is dependent on the value of the updated node (target), we need to use a different function for the interpretation of the PEW token.

## Example 1¶

In [3]:

```
from itertools import combinations, product
from collections import Counter

rules='''
x1*= [SDDS,0.1,0.8](x2)
x2*= [SDDS,0.5,0.1](x1 and not x2)
'''

model = b2p.Model(rules, mode='sync')

nodes=model.nodes.copy()
value_combinations=list(product([0.0,1.0],repeat=len(nodes)))

edge_occurances=Counter()
fp_state_dict={}
ens_size=1000
for ens in range(ens_size):
    for i in value_combinations:

        initial_state=dict(zip(nodes,i))
        model.initialize(lambda snode: initial_state[snode])
        model.iterate(1)#,shuffler=lambda lines: controlled_async_pick(lines,node))
        fp_state_dict=merge_two_dicts(fp_state_dict,dict(zip(model.fp(),model.states)))
        fp_states=model.fp()
        edge_occurances+=Counter([(fp_states[0],fp_states[-1])])
        
edge_probabilities={k:float(v)/(ens_size) for k,v in edge_occurances.items()}
for e,p in edge_probabilities.items():
    print('P(',fp_state_dict[e[0]].values(),'-->',fp_state_dict[e[1]].values(),')=',p)
```

```
P( [0.0, 0.0] --> [0.0, 0.0] )= 1.0
P( [0.0, 1.0] --> [0.0, 0.0] )= 0.82
P( [1.0, 0.0] --> [1.0, 0.0] )= 0.398
P( [1.0, 1.0] --> [1.0, 0.0] )= 0.901
P( [1.0, 0.0] --> [1.0, 1.0] )= 0.388
P( [1.0, 0.0] --> [0.0, 0.0] )= 0.113
P( [1.0, 1.0] --> [1.0, 1.0] )= 0.099
P( [0.0, 1.0] --> [0.0, 1.0] )= 0.082
P( [1.0, 0.0] --> [0.0, 1.0] )= 0.101
P( [0.0, 1.0] --> [1.0, 0.0] )= 0.088
P( [0.0, 1.0] --> [1.0, 1.0] )= 0.01
```

For reference the transition probabilities from the Murrugarra et al. paper.

In [ ]:

```

```
