# Supplementary material for: Probabilistic edge weights fine-tune Boolean network dynamics
Source: PLoS Comput Biol. 2022 Oct 10;18(10):e1010536. doi: 10.1371/journal.pcbi.1010536 (PMC9584532; doi:10.1371/journal.pcbi.1010536)
Supplement: S2 Notebook — (HTML) [file pcbi.1010536.s006.html]

Poret\_et\_al\_paper\_results-Boolean\_init


In [1]:

```
import random
import boolean2pew as b2p
import pandas as pd
import numpy as np
import EnsembleSimulation as es
```

In [2]:

```
def general_async_pick( lines ):
    line = [ random.choice( lines )]
    return line

def sigmoid(epsilon, x):
    return (1 / (1 + np.exp(-epsilon*(x))) )
```

## Reproducing the results of the Poret et al paper¶

Poret, A., Sousa, C. M., & Boissel, J. P. (2014). Enhancing Boolean networks with fuzzy operators and edge tuning.
https://hal.archives-ouvertes.fr/hal-01018236v4

For each simulation the first image is reproduced from the paper and the following plot is the reproduction of the qulaitative behavior with the PEW operators.

In [3]:

```
update_scheme_name='async'
update_mode='async'
shuffler=general_async_pick

initial_state={'EGF':0, 
               'HRG':0,
               'EGFR':0,
               'PI3K':0, 
               'AKT':0, 
               'Raf':0, 
               'ERK':0}
node_order=list(initial_state.keys())
break_state=[]

ensemble_size=1000
steps=200
end_EGF=50
```

## Simulation 1¶

In [4]:

```
rules = '''EGF*= EGF
HRG*= HRG
EGFR*= EGF or HRG
PI3K*= EGFR and not ERK
AKT*= PI3K
Raf*= EGFR or AKT
ERK*= Raf'''

end_EGF=steps
manipulation_set=[{'node':'EGF','enforced_state':1, 'start_time':20, 'end_time':end_EGF,'success_probability':1}]

model = b2p.Model(rules, mode=update_mode)

sim=es.EnsembleSimulation(model,shuffler=shuffler,ensemble_size=ensemble_size, steps=steps)
sim.simulate_manipulated_ensemble(manipulation_set)
sim.plot_node_evolution_averages(linewidth=2)
```

```
100%|██████████████████████████████████████| 1000/1000 [00:09<00:00, 103.09it/s]

Bad key "text.kerning_factor" on line 4 in
/home/david/anaconda3/envs/py3_env/lib/python3.7/site-packages/matplotlib/mpl-data/stylelib/_classic_test_patch.mplstyle.
You probably need to get an updated matplotlibrc file from
https://github.com/matplotlib/matplotlib/blob/v3.1.3/matplotlibrc.template
or from the matplotlib source distribution
```

## Simulation 2¶

In [5]:

```
rules = '''EGF*= EGF
HRG*= HRG
EGFR*= EGF or HRG
PI3K*= EGFR and [1,0.2]not ERK
AKT*= PI3K
Raf*= EGFR or AKT
ERK*= Raf'''

end_EGF=steps
manipulation_set=[{'node':'EGF','enforced_state':1, 'start_time':20, 'end_time':end_EGF,'success_probability':1}]

model = b2p.Model(rules, mode=update_mode)

sim=es.EnsembleSimulation(model,shuffler=shuffler,ensemble_size=ensemble_size, steps=steps)
sim.simulate_manipulated_ensemble(manipulation_set)
sim.plot_node_evolution_averages(linewidth=2)
```

```
100%|███████████████████████████████████████| 1000/1000 [00:10<00:00, 93.91it/s]
```

## Simulation 3¶

For this we would need an e vector to store the $e\_{ij}(k)$ values as well:
$e\_{ij}(k+1) = (1 - p\_{ij})\cdot e\_{ij}(k) + p\_{ij} \cdot x\_i(k)$
  
Here we add an extra node representing the edge, also using the addition "+" operator in the rule.

$e\_{ij}(k+1) = (1 - p\_{ij})\cdot e\_{ij}(k) + p\_{ij} \cdot x\_i(k)$

In [6]:

```
rules = '''EGF*= EGF
HRG*= HRG
EGFR*= EGF or HRG
PI3K*= EGFR and not e_ERK_PI3K
AKT*= PI3K
Raf*= EGFR or AKT
ERK*= Raf
e_ERK_PI3K*= (0.75 and e_ERK_PI3K) + (0.25 and ERK)'''


initial_state={'EGF':0, 
               'HRG':0,
               'EGFR':0,
               'PI3K':0, 
               'AKT':0, 
               'Raf':0, 
               'ERK':0,
               'e_ERK_PI3K':0}

end_EGF=steps
manipulation_set=[{'node':'EGF','enforced_state':1, 'start_time':20, 'end_time':end_EGF,'success_probability':1}]

model = b2p.Model(rules, mode=update_mode)

sim=es.EnsembleSimulation(model,shuffler=shuffler,ensemble_size=ensemble_size, steps=steps)
sim.simulate_manipulated_ensemble(manipulation_set)
sim.plot_node_evolution_averages(linewidth=2)
```

```
100%|███████████████████████████████████████| 1000/1000 [00:10<00:00, 91.27it/s]
```

## Simulation 4¶

In [7]:

```
rules = '''EGF*= [0.5,0.5](EGF)
HRG*= HRG
EGFR*= EGF or HRG
PI3K*= EGFR and not ERK
AKT*= PI3K
Raf*= EGFR or AKT
ERK*= Raf'''

initial_state={'EGF':0, 
               'HRG':0,
               'EGFR':0,
               'PI3K':0, 
               'AKT':0, 
               'Raf':0, 
               'ERK':0}
end_EGF=steps
manipulation_set=[{'node':'EGF','enforced_state':1, 'start_time':20, 'end_time':end_EGF,'success_probability':1}]

model = b2p.Model(rules, mode=update_mode)

sim=es.EnsembleSimulation(model,shuffler=shuffler,ensemble_size=ensemble_size, steps=steps)
sim.simulate_manipulated_ensemble([])
sim.plot_node_evolution_averages(linewidth=2)
```

```
100%|██████████████████████████████████████| 1000/1000 [00:08<00:00, 111.46it/s]
```

## Simulation 5¶

In [8]:

```
rules = ''' EGF*= [0.5,0.5] EGF
HRG*= HRG
EGFR*= EGF or HRG
PI3K*= EGFR and not e_ERK_PI3K
AKT*= PI3K
Raf*= EGFR or AKT
ERK*= Raf
e_ERK_PI3K*= (0.75 and e_ERK_PI3K) + (0.25 and ERK)'''

initial_state={'EGF':0, 
               'HRG':0,
               'EGFR':0,
               'PI3K':0, 
               'AKT':0, 
               'Raf':0, 
               'ERK':0,
               'e_ERK_PI3K':0}
end_EGF=steps
manipulation_set=[{'node':'EGF','enforced_state':1, 'start_time':20, 'end_time':end_EGF,'success_probability':1}]

model = b2p.Model(rules, mode=update_mode)

sim=es.EnsembleSimulation(model,shuffler=shuffler,ensemble_size=ensemble_size, steps=steps)
sim.simulate_manipulated_ensemble(manipulation_set=[])
sim.plot_node_evolution_averages(linewidth=2)
```

```
100%|███████████████████████████████████████| 1000/1000 [00:10<00:00, 97.50it/s]
```

In [ ]:

```

```
