# Supplementary material for: Probabilistic edge weights fine-tune Boolean network dynamics
Source: PLoS Comput Biol. 2022 Oct 10;18(10):e1010536. doi: 10.1371/journal.pcbi.1010536 (PMC9584532; doi:10.1371/journal.pcbi.1010536)
Supplement: S3 Notebook — (HTML) [file pcbi.1010536.s007.html]

Supplementary\_Application\_Note


In [1]:

```
import random
import boolean2pew as b2p
from PEW_functions import *
def general_async_pick( lines ):
    line = [ random.choice( lines )]
    return line
```

! For the general PEW functions see PEW\_functions.py

# 1. Doubly Tyr phosphorylated p27Kip1 partially reactivates Cdk4/CyclinD¶

Experimental paper: Ou, L., Waddell, M. B., & Kriwacki, R. W. (2012). Mechanism of cell cycle entry mediated by the intrinsically disordered protein p27Kip1. ACS chemical biology, 7(4), 678-682.

In [6]:

```
rules='''Cdk4*= not p27 and not p27_tyr88 and [1,0.2] not p27_tyr88_tyr74
p27 *= NRTK
p27_tyr88 *= p27 and (Abl or Src)
p27_tyr88_tyr74 *= p27_tyr88_tyr74 or (p27_tyr88 and Src)
'''

initial_state={'Cdk4':1,'p27':0,'p27_tyr88':0,'p27_tyr88_tyr74':0, 'NRTK':0, 'Src':0, 'Abl':0}   

model = b2p.Model(rules, mode='async')
model.initialize(lambda node: initial_state[node])
import EnsembleSimulation as esim
update_scheme_name='async'
update_mode='async'
shuffler=general_async_pick
  
node_order=list(initial_state.keys())
break_states=[]
ensemble_size=500
steps=250

c_avg_dict={}
manipulation_set=[{'node':'NRTK','enforced_state':1, 'start_time':40, 'end_time':80,'success_probability':1},
                  {'node':'NRTK','enforced_state':0, 'start_time':160, 'end_time':200,'success_probability':1},
                  {'node':'Abl','enforced_state':1, 'start_time':81, 'end_time':120,'success_probability':1},
                  {'node':'Src','enforced_state':1, 'start_time':121, 'end_time':200,'success_probability':1}]


model = b2p.Model(rules, mode=update_mode)
es=esim.EnsembleSimulation(model,initial_state=initial_state,ensemble_size=ensemble_size,steps=steps, shuffler=shuffler)
es.simulate_manipulated_ensemble(manipulation_set,break_states=break_states)

#monitored_nodes=model.nodes
monitored_nodes=['Cdk4','p27','NRTK','p27_tyr88','p27_tyr88_tyr74','Abl','Src']
es.plot_node_evolution_averages(nodes=monitored_nodes, figsize=(10,6),linewidth=2)
```

```
100%|█████████████████████████████████████████| 500/500 [00:09<00:00, 52.97it/s]
```

# 2. E2F hysteresis is reproduced by PEW operator combined with SDDS function¶

Experimental paper: Yao, G., Lee, T. J., Mori, S., Nevins, J. R., & You, L. (2008). A bistable Rb–E2F switch underlies the restriction point. Nature cell biology, 10(4), 476-482.

### With GF pulse:¶

In [3]:

```
rules='''CyclinD*= (Myc and GF) 
CyclinE*= E2F
E2F*= [SDDS,1,0.99] ((not RB) and Myc)
Myc*= GF
RB*= (not CyclinD) and (not CyclinE)'''

initial_state={'GF':1,'CyclinD':0,'Myc':1,'CyclinE':0,'E2F':0,'RB':1}   

model = b2p.Model(rules, mode='async')
model.initialize(lambda node: initial_state[node])
model.iterate(steps, shuffler=general_async_pick)
import EnsembleSimulation as esim
update_scheme_name='async'
update_mode='async'
shuffler=general_async_pick
  
node_order=list(initial_state.keys())
break_states=[]
ensemble_size=500
steps=100

c_avg_dict={}
manipulation_set=[{'node':'GF','enforced_state':1, 'start_time':20, 'end_time':100,'success_probability':1},
                 {'node':'GF','enforced_state':0, 'start_time':20, 'end_time':100,'success_probability':0.8}]


model = b2p.Model(rules, mode=update_mode)
es=esim.EnsembleSimulation(model,initial_state=initial_state,ensemble_size=ensemble_size,steps=steps, shuffler=shuffler)
es.simulate_manipulated_ensemble(manipulation_set,break_states=break_states)

monitored_nodes=model.nodes
es.plot_node_evolution_averages(nodes=monitored_nodes, figsize=(10,6),linewidth=2)
```

```
100%|█████████████████████████████████████████| 500/500 [00:07<00:00, 63.93it/s]
```

### No GF pulse¶

In [4]:

```
rules='''CyclinD*= (Myc and GF) 
CyclinE*= E2F
E2F*= [SDDS,1,0.99] ((not RB) and Myc)
Myc*= GF
RB*= (not CyclinD) and (not CyclinE)'''

initial_state={'GF':1,'CyclinD':0,'Myc':0,'CyclinE':0,'E2F':0,'RB':1}      

model = b2p.Model(rules, mode='async')
model.initialize(lambda node: initial_state[node])
model.iterate(steps, shuffler=general_async_pick)
import EnsembleSimulation as esim
update_scheme_name='async'
update_mode='async'
shuffler=general_async_pick
  
node_order=list(initial_state.keys())
break_states=[]
ensemble_size=500
steps=100

c_avg_dict={}
manipulation_set=[{'node':'GF','enforced_state':1, 'start_time':0, 'end_time':100,'success_probability':1},
                 {'node':'GF','enforced_state':0, 'start_time':0, 'end_time':100,'success_probability':0.8}]


model = b2p.Model(rules, mode=update_mode)
es=esim.EnsembleSimulation(model,initial_state=initial_state,ensemble_size=ensemble_size,steps=steps, shuffler=shuffler)
es.simulate_manipulated_ensemble(manipulation_set,break_states=break_states)

monitored_nodes=model.nodes
es.plot_node_evolution_averages(nodes=monitored_nodes, figsize=(10,6),linewidth=2)
```

```
100%|█████████████████████████████████████████| 500/500 [00:07<00:00, 62.89it/s]
```

# 3. Exponential noise function produces sophisticated node behaviors¶

In [5]:

```
rules='''A*=[exponential,0.1,0.1] B
C*=[exponential,0.15,0.15] B
D*=[exponential,0.2,0.2] B
E*=[exponential,0.25,0.25] B'''
ensemble_size=500
steps=200

initial_state={'A':0,'B':1,'C':0,'D':0, 'E':0}   
model = b2p.Model(rules, mode='async')
model.initialize(lambda node: initial_state[node])
model.iterate(steps, shuffler=general_async_pick)
import EnsembleSimulation as esim
update_scheme_name='async'
update_mode='async'
shuffler=general_async_pick
  
node_order=list(initial_state.keys())
break_states=[]


c_avg_dict={}
manipulation_set=[{'node':'B','enforced_state':1, 'start_time':30, 'end_time':60,'success_probability':1},
                 {'node':'B','enforced_state':0, 'start_time':60, 'end_time':100,'success_probability':1}]


model = b2p.Model(rules, mode=update_mode)
es=esim.EnsembleSimulation(model,initial_state=initial_state,ensemble_size=ensemble_size,steps=steps, shuffler=shuffler)
es.simulate_manipulated_ensemble(manipulation_set,break_states=break_states)

monitored_nodes=model.nodes
es.plot_node_evolution_averages(nodes=monitored_nodes, figsize=(10,6),linewidth=2)
```

```
100%|█████████████████████████████████████████| 500/500 [00:46<00:00, 10.67it/s]
```
