# Supplementary material for: Probabilistic edge weights fine-tune Boolean network dynamics
Source: PLoS Comput Biol. 2022 Oct 10;18(10):e1010536. doi: 10.1371/journal.pcbi.1010536 (PMC9584532; doi:10.1371/journal.pcbi.1010536)
Supplement: S4 Notebook — (HTML) [file pcbi.1010536.s008.html]

Applications


# Probabilistic Edge Weights fine-tune Boolean network dynamics¶

In [2]:

```
import EnsembleSimulation as esim
import boolean2pew as b2p
import numpy as np
import matplotlib.pyplot as plt
%matplotlib inline
import seaborn as sns
import random

import pandas as pd
from collections import Counter
```

# Noisy feedback-loops explain the loss of M attractor stability in epithelial to mesenchymal transition (EMT)¶

Celia-Terrasa et al paper with the original experiments and analysis: https://www.nature.com/articles/s41467-018-07538-7

In [3]:

```
def general_async_pick( lines ):
    line = [ random.choice( lines )]
    #print data
    return line


update_scheme_name='async'
update_mode='async'
shuffler=general_async_pick

initial_state={'TGFB':0, 
               'minter1':0,
               'minter2':0,
               'SMAD':0, 
               'ZEB1':0, 
               'miR200':1, 
               'inter1':0,
               'inter2':0,
               'inter3':0,
               'inter4':0,
               'inter5':0,
               'inter6':0,
               'SNAI1':0,
               'Ecadherin':1}
node_order=list(initial_state.keys())
break_states=[]
ensemble_size=200
steps=2000
```

In [4]:

```
rules = '''TGFB *= TGFB
SMAD *= TGFB or ZEB1
ZEB1 *= SMAD and not miR200
minter1 *= TGFB
minter2 *= minter1
miR200 *= [1,0.05](not ZEB1) and not SNAI1 and not minter2
inter1 *= TGFB
inter2 *= inter1
inter3 *= inter2
inter4 *= inter3
inter5 *= inter4
inter6 *= inter5 or [0.9,0]SNAI1
SNAI1 *= inter6
Ecadherin *= not SNAI1 and not ZEB1'''

p_TGFB_range=np.linspace(0,0.2,5)

model_name='EMT_alt'
p_TGFB=1
stop_TGFB=50
end_TGFB=stop_TGFB
ec_avg_dict={}
for p_TGFB in p_TGFB_range:
    manipulation_set=[#{'node':'TGFB','enforced_state':0, 'start_time':1, 'end_time':end_TGFB,'success_probability':1},
                      {'node':'TGFB','enforced_state':1, 'start_time':1, 'end_time':end_TGFB,'success_probability':p_TGFB},
                      {'node':'TGFB','enforced_state':0, 'start_time':end_TGFB, 'end_time':end_TGFB+1,'success_probability':1}]#,

    model = b2p.Model(rules, mode=update_mode)
    es=esim.EnsembleSimulation(model,initial_state,shuffler=shuffler,ensemble_size=ensemble_size,steps=steps)
    es.simulate_manipulated_ensemble(manipulation_set,break_states=break_states)
    
    timeslice_start=100
    timeslice_end=150
    df=es.average_state_of_time_slice(timeslice_start,timeslice_end)

    ec_avg_dict[p_TGFB]=df.Ecadherin
    df_final_states=pd.DataFrame(es.final_states)

    print(Counter(df_final_states['Ecadherin']))
    
    es.plot_node_evolution_averages(nodes=['TGFB','ZEB1','SNAI1','Ecadherin'], figsize=(10,6), linewidth=2)
```

```
100%|█████████████████████████████████████████| 200/200 [00:25<00:00,  7.86it/s]
```

```
Counter({1.0: 200})
```

```
100%|█████████████████████████████████████████| 200/200 [00:25<00:00,  7.92it/s]
```

```
Counter({1.0: 195, 0.0: 5})
```

```
100%|█████████████████████████████████████████| 200/200 [00:24<00:00,  8.13it/s]
```

```
Counter({1.0: 197, 0.0: 3})
```

```
100%|█████████████████████████████████████████| 200/200 [00:25<00:00,  7.77it/s]
```

```
Counter({1.0: 197, 0.0: 3})
```

```
100%|█████████████████████████████████████████| 200/200 [00:24<00:00,  8.06it/s]
```

```
Counter({1.0: 193, 0.0: 7})
```

In [3]:

```
es.plot_node_evolution_averages(nodes=['TGFB','ZEB1','SNAI1','Ecadherin'], figsize=(10,6), linewidth=2, title='Wild type',save_path='figures/EMT_wildtype.svg')

medians_wt=[]
std_wt=[]
fig, axs = plt.subplots(len(ec_avg_dict), sharex=True, sharey=False, figsize=(10,12))
fig.suptitle('Wild type', fontsize=24)
for  i,TGFB_end in enumerate(sorted(ec_avg_dict)):
    axs[i].hist(ec_avg_dict[TGFB_end], bins=np.linspace(-0.1,1.1,20))
    max_y=np.histogram(ec_avg_dict[TGFB_end], bins=np.linspace(-0.1,1.1,20))[0].max()
    axs[i].grid()
    axs[i].vlines(np.median(ec_avg_dict[TGFB_end]), 0, max_y, colors='red', linestyles='solid',label='median=%f'%np.median(ec_avg_dict[TGFB_end]),linewidth=2)
    axs[i].set_title('Pr(TGFB=1) = %f'%TGFB_end)
    medians_wt.append(np.median(ec_avg_dict[TGFB_end]))
    std_wt.append(np.std(ec_avg_dict[TGFB_end]))
    
fig.text(0.5, 0.04, 'Ensemble avg of E-cadherin between steps 100 to 150', ha='center', fontsize=20)
fig.text(0.04, 0.5, 'Count of nodes in each bin', va='center', rotation='vertical', fontsize=20)
plt.savefig('figures/EMT_tgfb_conc_wildtype.svg')
```

In [23]:

```
rules = '''TGFB *= TGFB
SMAD *= TGFB or ZEB1
ZEB1 *= SMAD and not miR200
minter1 *= TGFB
minter2 *= minter1
miR200 *= [1,0.99](not ZEB1) and not SNAI1 and not minter2
inter1 *= TGFB
inter2 *= inter1
inter3 *= inter2
inter4 *= inter3
inter5 *= inter4
inter6 *= inter5 or [0.9,0] SNAI1
SNAI1 *= inter6
Ecadherin *= not SNAI1 and not ZEB1'''

model_name='EMT_alt'
p_TGFB=1
stop_TGFB=50
end_TGFB=stop_TGFB
ec_avg_dict={}
for p_TGFB in p_TGFB_range:
    manipulation_set=[#{'node':'TGFB','enforced_state':0, 'start_time':1, 'end_time':end_TGFB,'success_probability':1},
                      {'node':'TGFB','enforced_state':1, 'start_time':1, 'end_time':end_TGFB,'success_probability':p_TGFB},
                      {'node':'TGFB','enforced_state':0, 'start_time':end_TGFB, 'end_time':end_TGFB+1,'success_probability':1}]#,

    model = b2p.Model(rules, mode=update_mode)
    es=esim.EnsembleSimulation(model,initial_state,shuffler=shuffler,ensemble_size=ensemble_size,steps=steps)
    es.simulate_manipulated_ensemble(manipulation_set,break_states=break_states)
    
    timeslice_start=100
    timeslice_end=150
    df=es.average_state_of_time_slice(timeslice_start,timeslice_end)

    ec_avg_dict[p_TGFB]=df.Ecadherin
    df_final_states=pd.DataFrame(es.final_states)

    print(Counter(df_final_states['Ecadherin']))
    
    es.plot_node_evolution_averages(nodes=['TGFB','ZEB1','SNAI1','Ecadherin'], figsize=(10,6), linewidth=2)
```

```
100%|█████████████████████████████████████████| 200/200 [00:23<00:00,  8.46it/s]
```

```
Counter({1.0: 200})
```

```
100%|█████████████████████████████████████████| 200/200 [00:23<00:00,  8.38it/s]
```

```
Counter({1.0: 200})
```

```
100%|█████████████████████████████████████████| 200/200 [00:23<00:00,  8.47it/s]
```

```
Counter({1.0: 200})
```

```
100%|█████████████████████████████████████████| 200/200 [00:22<00:00,  8.73it/s]
```

```
Counter({1.0: 200})
```

```
100%|█████████████████████████████████████████| 200/200 [00:24<00:00,  8.27it/s]
```

```
Counter({1.0: 199, 0.0: 1})
```

In [24]:

```
es.plot_node_evolution_averages(nodes=['TGFB','ZEB1','SNAI1','Ecadherin'], figsize=(10,6), linewidth=2, title='Mutant',save_path='figures/EMT_mutant.svg')
medians_mutant=[]
std_mutant=[]
fig, axs = plt.subplots(len(ec_avg_dict), sharex=True, sharey=False, figsize=(10,12))
fig.suptitle('Mutant', fontsize=24)
for  i,TGFB_end in enumerate(sorted(ec_avg_dict)):
    axs[i].hist(ec_avg_dict[TGFB_end], bins=np.linspace(-0.1,1.1,20))
    max_y=np.histogram(ec_avg_dict[TGFB_end], bins=np.linspace(-0.1,1.1,20))[0].max()
    axs[i].grid()
    axs[i].vlines(np.median(ec_avg_dict[TGFB_end]), 0, max_y, colors='red', linestyles='solid',label='median=%f'%np.median(ec_avg_dict[TGFB_end]),linewidth=2)
    axs[i].set_title('Pr(TGFB=1) = %f'%TGFB_end)
    medians_mutant.append(np.median(ec_avg_dict[TGFB_end]))
    std_mutant.append(np.std(ec_avg_dict[TGFB_end]))
    
fig.text(0.5, 0.04, 'Ensemble avg of E-cadherin between steps 100 to 150', ha='center', fontsize=20)
fig.text(0.04, 0.5, 'Count of nodes in each bin', va='center', rotation='vertical', fontsize=20)
plt.savefig('figures/EMT_tgfb_conc_mutant.svg')
```

In [ ]:

```
plt.figure()
plt.errorbar(p_TGFB_range,medians_mutant, yerr=np.array(std_mutant)/np.sqrt(ensemble_size), label='mutant')
plt.errorbar(p_TGFB_range,medians_wt, yerr=np.array(std_wt)/np.sqrt(ensemble_size), label='wild type')
plt.legend()
```

# Reduced CyclinB-Cdk1 induced apoptosis improves asynchronous cell cycle model¶

This section of the notebook contains the simulations and analysis describes in the second application of the paper.

Sizek et al paper: https://journals.plos.org/ploscompbiol/article?id=10.1371/journal.pcbi.1006402

In [7]:

```
update_scheme_name='async'
update_mode='async'
shuffler=general_async_pick
init_array=np.loadtxt('cell_cycle_init_G0.txt',dtype=str)
initial_state=dict(zip(init_array[:,0],init_array[:,1].astype(bool)))
node_order=list(initial_state.keys())
break_states=[]
ensemble_size=100
steps=10000
```

In [8]:

```
monitored_nodes=['GF_High','CyclinA','CyclinB','Cdk1','Casp3','Casp2' ,'MCL_1','BCLXL','BCL2','CAD']
```

# Original Sizek et al. model¶

In [9]:

```
rules = '''GF *= GF or GF_High
RTK *= not CAD and (GF_High or GF)
Grb2 *= RTK and GF_High
Ras *= Grb2 and SOS
RAF *= not Casp3 and Ras
mTORC2 *= PIP3 or not S6K
PI3K *= Ras or RTK
PIP3 *= PI3K_H or PI3K
PDK1 *= PI3K and PIP3
AKT_B *= not Casp3 and PIP3 and (PDK1 or mTORC2)
p110_H *= (FoxO3 and not NeddL4) or (p110_H and (FoxO3 or not NeddL4) )
PI3K_H *= p110_H and RTK and PI3K and Ras
AKT_H *= AKT_B and p110_H and PI3K_H and Ras and PIP3 and PDK1 and mTORC2
FoxO3 *= not (AKT_B or AKT_H or ERK) or ( not (AKT_H and (Plk1 or Plk1_H or AKT_B or ERK) ) and not (Plk1 and Plk1_H and ERK) )
PLCgamma *= RTK and Grb2 and p110_H and PI3K_H and PIP3
NeddL4 *= Ca2 and IP3
FoxO1 *= not Plk1 and not AKT_H
p21_mRNA *= (FoxO1 and FoxO3) or (not Myc and (FoxO1 or FoxO3) )
TSC2 *= not AKT_H or not (AKT_B or ERK)
PRAS40 *= not AKT_H and (not mTORC1 or not AKT_B)
Rheb *= not TSC2 and DAG
mTORC1 *= not Casp3 and ( (Rheb and not PRAS40) or E2F1 or (CyclinB and Cdk1 and GSK3) )
S6K *= not Casp3 and mTORC1
eIF4E *= not Casp3 and mTORC1
GSK3 *=  not AKT_H and not (S6K and ERK)
p21 *= p21_mRNA and not Casp3 and not CyclinE
pRB *= not Casp3 and not CyclinD1 and not CyclinA and (p27Kip1 or not CyclinE)
p27Kip1 *= not Casp3 and not CyclinD1 and not (Cdk1 and CyclinB) and ( (not CyclinE and (FoxO3 and FoxO1) ) or (not CyclinA and (FoxO3 or FoxO1) ) or (not CyclinE and not CyclinA) )
Myc *= (ERK and (eIF4E or not GSK3) ) or ( E2F1 and not pRB and (eIF4E or ERK or not GSK3) )
CyclinD1 *= not CHK1 and ( ( not p21 and ( (not GSK3 and (Myc or E2F1) ) or (Myc and CyclinD1) or (Myc and E2F1) or (E2F1 and CyclinD1) ) ) or ( not pRB and E2F1 and ( (Myc and CyclinD1) or (Myc and not GSK3) or (CyclinD1 and not GSK3) ) ) )
E2F1 *= not (CAD or CyclinA or pRB) and (E2F1 or Myc)
CyclinE *= E2F1 and Cdc6 and Pre_RC and not (pRB or p27Kip1 or CHK1 or Casp3)
ORC *= E2F1 or (Pre_RC and Cdt1 and Cdc6)
Cdc6 *= not Casp3 and not (f4N_DNA and CyclinA) and ( (E2F1 and ORC and not Plk1) or (Pre_RC and ORC and Cdc6 and Cdt1) )
Cdt1 *= not geminin and ORC and Cdc6 and not (CyclinE and CyclinA and Cdc25A) and ( (Pre_RC and (E2F1 or Myc) ) or (E2F1 and (Myc or not pRB) ) )
Pre_RC *= ORC and Cdc6 and Cdt1 and not (Replication and f4N_DNA)
geminin *= E2F1 and not Cdh1 and not (pAPC and Cdc20)
CyclinA_mRNA *= not CAD and ( (E2F1 and not pRB) or FoxM1 )
Emi1 *= (E2F1 or not pRB or not p21) and not (Plk1 and CyclinB and Cdk1 and (U_Kinetochores or A_Kinetochores) )
FoxM1 *= (Myc and CyclinE) or (CyclinA and Cdc25A and Cdc25B) or (Plk1 and CyclinB and Cdk1)
Cdc25A *= ( (FoxM1 and E2F1 and not pRB) or (not Cdh1 and (FoxM1 or (E2F1 and not pRB) ) ) ) and (not (GSK3 or CHK1) or CyclinE or CyclinA or (CyclinB and Cdk1) )                   
CyclinA *= CyclinA_mRNA and not pAPC and ( (Cdc25A and (not Cdh1 or Emi1) ) or (CyclinA and ( (not Cdh1 and (Emi1 or not UbcH10) ) or (Emi1 and not UbcH10) ) ) )
Wee1 *= not Casp3 and not (Cdk1 and CyclinB) and (Replication or CHK1) and (CHK1 or not (Cdk1 and CyclinA and Plk1) )
UbcH10 *= not Cdh1 or (UbcH10 and (Cdc20 or CyclinA or CyclinB) )
CyclinB *= (FoxM1 or (FoxO3 and CyclinB) ) and not (Cdh1 or (pAPC and Cdc20) )
Cdc25B *= FoxM1 and f4N_DNA
Plk1 *= not Cdh1 and (FoxM1 or Plk1_H) and ( (CyclinB and Cdk1) or (CyclinA and not Wee1 and Cdc25A) )
Cdc25C *= f4N_DNA and Plk1 and ( (Cdc25B and not CHK1) or (CyclinB and Cdk1) )
Cdk1 *= CyclinB and Cdc25C and ( not CHK1 or (not Wee1 and Cdk1) )
pAPC *= (CyclinB and Cdk1 and Plk1) or (CyclinB and Cdk1 and pAPC) or (pAPC and Cdc20)
Cdc20 *= pAPC and not Emi1 and not Cdh1 and ( not Mad2 or ( not CyclinA and not (CyclinB and Cdk1) ) )
Cdh1 *= not (CyclinB and Cdk1) and not ( CyclinA and (Emi1 or Cdc25A) )
Replication *= not CAD and Pre_RC and ( (E2F1 and CyclinE and Cdc25A) or (Replication and CyclinA and Cdc25A and (E2F1 or not f4N_DNA) ) )
f4N_DNA *= not CAD and ( (Replication and ( (Pre_RC and CyclinA) or f4N_DNA) ) or (f4N_DNA and not Ect2) )
U_Kinetochores *= f4N_DNA and not Cdh1 and not A_Kinetochores and ( (CyclinB and Cdk1) or U_Kinetochores )
Mad2 *= U_Kinetochores and not A_Kinetochores
A_Kinetochores *= f4N_DNA and not Cdh1 and not (pAPC and Cdc20) and ( A_Kinetochores or (U_Kinetochores and Plk1 and CyclinB and Cdk1) )
Plk1_H *= Plk1 and FoxM1 and (Plk1_H or FoxO3 or FoxO1)
Ect2 *= f4N_DNA and Plk1_H and Cdh1 and not U_Kinetochores and not A_Kinetochores
Casp8 *= DR4_5 or Casp3
Casp2 *= Casp3 or (U_Kinetochores and Mad2 and not (CyclinB and Cdk1) )
MCL_1 *= not Casp3 and not Casp2 and (not GSK3 or (AKT_B and (ERK or not E2F1) ) ) and (not (Cdk1 and CyclinB and U_Kinetochores) )
BCLXL *= not Casp3 and (BCL2 or not BAD) and ( not U_Kinetochores or ( Plk1 and ( not (CyclinB and Cdk1) or (BCL2 and MCL_1) ) ) or  ( (BCL2 and MCL_1) and not (CyclinB and Cdk1) ) )
BCL2 *= not (Casp3 or BAD or BIM or BIK) and  ( not U_Kinetochores or (MCL_1 and BCLXL) or (Plk1 and (BCLXL or MCL_1 or not (Cdk1 and CyclinB) ) ) )
BAD *= Casp3 or not (AKT_H or AKT_B or ERK or S6K) or ( Casp8 and (not (AKT_B and ERK and S6K) and not (AKT_H and (AKT_B or ERK) ) ) )
BIK *= not (MCL_1 or BCLXL or BCL2)
BIM *= FoxO3 and GSK3 and not (ERK or MCL_1 or BCLXL or BCL2)
BID *= Casp8 or ( Casp2 and not (BCL2 or BCLXL or MCL_1) )
BAK *= (BID and (BIM or BIK or not (BCL2 and BCLXL and MCL_1) ) ) or ( (BIM or BIK) and not (BCLXL or MCL_1) )
BAX *= (BIM and ( (BID or BIK) or not (BCL2 and BCLXL and MCL_1) ) ) or ( (BID or BIK) and not (BCL2 or BCLXL) )
Cyto_C *= BAX or BAK
SMAC *= BAX or BAK
IAPs *= not SMAC or AKT_H
Casp9 *= (Casp3 or (not IAPs and Cyto_C))
Casp3 *= (Casp9 and Casp8) or ( Casp3 and (Casp9 or Casp8) ) or ( not IAPs and (Casp9 or Casp8 or Casp3) )
CAD *= Casp3 and Casp9
ATR *= Replication
CHK1 *= ATR
Ca2 *= IP3
DAG *= PLCgamma
DR4_5 *= Trail
ERK *= MEK and not BIK
GF_High *= GF_High
IP3 *= PLCgamma
MEK *= RAF
SOS *= Grb2
Trail *= Trail'''

model_name='CC'

ec_avg_dict={}
manipulation_set=[{'node':'GF_High','enforced_state':1, 'start_time':1, 'end_time':2,'success_probability':1}]
                 #{'node':'GF_High','enforced_state':0, 'start_time':3001, 'end_time':steps,'success_probability':0}]

model = b2p.Model(rules, mode=update_mode)
es=esim.EnsembleSimulation(model,initial_state=initial_state,shuffler=shuffler,ensemble_size=ensemble_size,steps=steps)
es.simulate_manipulated_ensemble(manipulation_set,break_states=break_states)

timeslice_start=0
timeslice_end=steps
df=es.average_state_of_time_slice(timeslice_start,timeslice_end)

df_final_states=pd.DataFrame(es.final_states)
```

```
100%|█████████████████████████████████████████| 100/100 [03:09<00:00,  1.90s/it]
```

In [11]:

```
es.plot_node_evolution_averages(nodes=monitored_nodes, figsize=(10,6), title='Original Sizek et al. Cell Cycle model',linewidth=2,save_path='figures/Sizek_original.svg')
```

# Reduced edge-weight of Cdk1-CyclinB on anti-apoptotic nodes¶

In [12]:

```
rules = '''GF *= GF or GF_High
RTK *= not CAD and (GF_High or GF)
Grb2 *= RTK and GF_High
Ras *= Grb2 and SOS
RAF *= not Casp3 and Ras
mTORC2 *= PIP3 or not S6K
PI3K *= Ras or RTK
PIP3 *= PI3K_H or PI3K
PDK1 *= PI3K and PIP3
AKT_B *= not Casp3 and PIP3 and (PDK1 or mTORC2)
p110_H *= (FoxO3 and not NeddL4) or (p110_H and (FoxO3 or not NeddL4) )
PI3K_H *= p110_H and RTK and PI3K and Ras
AKT_H *= AKT_B and p110_H and PI3K_H and Ras and PIP3 and PDK1 and mTORC2
FoxO3 *= not (AKT_B or AKT_H or ERK) or ( not (AKT_H and (Plk1 or Plk1_H or AKT_B or ERK) ) and not (Plk1 and Plk1_H and ERK) )
PLCgamma *= RTK and Grb2 and p110_H and PI3K_H and PIP3
NeddL4 *= Ca2 and IP3
FoxO1 *= not Plk1 and not AKT_H
p21_mRNA *= (FoxO1 and FoxO3) or (not Myc and (FoxO1 or FoxO3) )
TSC2 *= not AKT_H or not (AKT_B or ERK)
PRAS40 *= not AKT_H and (not mTORC1 or not AKT_B)
Rheb *= not TSC2 and DAG
mTORC1 *= not Casp3 and ( (Rheb and not PRAS40) or E2F1 or (CyclinB and Cdk1 and GSK3) )
S6K *= not Casp3 and mTORC1
eIF4E *= not Casp3 and mTORC1
GSK3 *=  not AKT_H and not (S6K and ERK)
p21 *= p21_mRNA and not Casp3 and not CyclinE
pRB *= not Casp3 and not CyclinD1 and not CyclinA and (p27Kip1 or not CyclinE)
p27Kip1 *= not Casp3 and not CyclinD1 and not (Cdk1 and CyclinB) and ( (not CyclinE and (FoxO3 and FoxO1) ) or (not CyclinA and (FoxO3 or FoxO1) ) or (not CyclinE and not CyclinA) )
Myc *= (ERK and (eIF4E or not GSK3) ) or ( E2F1 and not pRB and (eIF4E or ERK or not GSK3) )
CyclinD1 *= not CHK1 and ( ( not p21 and ( (not GSK3 and (Myc or E2F1) ) or (Myc and CyclinD1) or (Myc and E2F1) or (E2F1 and CyclinD1) ) ) or ( not pRB and E2F1 and ( (Myc and CyclinD1) or (Myc and not GSK3) or (CyclinD1 and not GSK3) ) ) )
E2F1 *= not (CAD or CyclinA or pRB) and (E2F1 or Myc)
CyclinE *= E2F1 and Cdc6 and Pre_RC and not (pRB or p27Kip1 or CHK1 or Casp3)
ORC *= E2F1 or (Pre_RC and Cdt1 and Cdc6)
Cdc6 *= not Casp3 and not (f4N_DNA and CyclinA) and ( (E2F1 and ORC and not Plk1) or (Pre_RC and ORC and Cdc6 and Cdt1) )
Cdt1 *= not geminin and ORC and Cdc6 and not (CyclinE and CyclinA and Cdc25A) and ( (Pre_RC and (E2F1 or Myc) ) or (E2F1 and (Myc or not pRB) ) )
Pre_RC *= ORC and Cdc6 and Cdt1 and not (Replication and f4N_DNA)
geminin *= E2F1 and not Cdh1 and not (pAPC and Cdc20)
CyclinA_mRNA *= not CAD and ( (E2F1 and not pRB) or FoxM1 )
Emi1 *= (E2F1 or not pRB or not p21) and not (Plk1 and CyclinB and Cdk1 and (U_Kinetochores or A_Kinetochores) )
FoxM1 *= (Myc and CyclinE) or (CyclinA and Cdc25A and Cdc25B) or (Plk1 and CyclinB and Cdk1)
Cdc25A *= ( (FoxM1 and E2F1 and not pRB) or (not Cdh1 and (FoxM1 or (E2F1 and not pRB) ) ) ) and (not (GSK3 or CHK1) or CyclinE or CyclinA or (CyclinB and Cdk1) )                   
CyclinA *= CyclinA_mRNA and not pAPC and ( (Cdc25A and (not Cdh1 or Emi1) ) or (CyclinA and ( (not Cdh1 and (Emi1 or not UbcH10) ) or (Emi1 and not UbcH10) ) ) )
Wee1 *= not Casp3 and not (Cdk1 and CyclinB) and (Replication or CHK1) and (CHK1 or not (Cdk1 and CyclinA and Plk1) )
UbcH10 *= not Cdh1 or (UbcH10 and (Cdc20 or CyclinA or CyclinB) )
CyclinB *= (FoxM1 or (FoxO3 and CyclinB)) and not (Cdh1 or (pAPC and Cdc20) )
Cdc25B *= FoxM1 and f4N_DNA
Plk1 *= not Cdh1 and (FoxM1 or Plk1_H) and ( (CyclinB and Cdk1) or (CyclinA and not Wee1 and Cdc25A) )
Cdc25C *= f4N_DNA and Plk1 and ( (Cdc25B and not CHK1) or (CyclinB and Cdk1) )
Cdk1 *= CyclinB and Cdc25C and ( not CHK1 or (not Wee1 and Cdk1) )
pAPC *= (CyclinB and Cdk1 and Plk1) or (CyclinB and Cdk1 and pAPC) or (pAPC and Cdc20)
Cdc20 *= pAPC and not Emi1 and not Cdh1 and ( not Mad2 or ( not CyclinA and not (CyclinB and Cdk1) ) )
Cdh1 *= not (CyclinB and Cdk1) and not ( CyclinA and (Emi1 or Cdc25A) )
Replication *= not CAD and Pre_RC and ( (E2F1 and CyclinE and Cdc25A) or (Replication and CyclinA and Cdc25A and (E2F1 or not f4N_DNA) ) )
f4N_DNA *= not CAD and ( (Replication and ( (Pre_RC and CyclinA) or f4N_DNA) ) or (f4N_DNA and not Ect2) )
U_Kinetochores *= f4N_DNA and not Cdh1 and not A_Kinetochores and ( (CyclinB and Cdk1) or U_Kinetochores )
Mad2 *= U_Kinetochores and not A_Kinetochores
A_Kinetochores *= f4N_DNA and not Cdh1 and not (pAPC and Cdc20) and ( A_Kinetochores or (U_Kinetochores and Plk1 and CyclinB and Cdk1) )
Plk1_H *= Plk1 and FoxM1 and (Plk1_H or FoxO3 or FoxO1)
Ect2 *= f4N_DNA and Plk1_H and Cdh1 and not U_Kinetochores and not A_Kinetochores
Casp8 *= DR4_5 or Casp3
Casp2 *= Casp3 or (U_Kinetochores and Mad2 and not (CyclinB and Cdk1) )
MCL_1 *= (not Casp3 and not Casp2 and ((not GSK3) or (AKT_B and (ERK or not E2F1) ) ) and (not ([0.5,0](Cdk1 and CyclinB) and U_Kinetochores) ))
BCLXL *= (not Casp3 and (BCL2 or not BAD) and ( not U_Kinetochores or ( Plk1 and ( not [0.5,0](CyclinB and Cdk1) or (BCL2 and MCL_1) ) ) or  ( (BCL2 and MCL_1) and not [0.5,0] (CyclinB and Cdk1) ) ))
BCL2 *= (not (Casp3 or BAD or BIM or BIK) and  ( not U_Kinetochores or (MCL_1 and BCLXL) or (Plk1 and (BCLXL or MCL_1 or not [0.5,0] (Cdk1 and CyclinB) ) ) ))
BAD *= Casp3 or not (AKT_H or AKT_B or ERK or S6K) or ( Casp8 and (not (AKT_B and ERK and S6K) and not (AKT_H and (AKT_B or ERK) ) ) )
BIK *= not (MCL_1 or BCLXL or BCL2)
BIM *= FoxO3 and GSK3 and not (ERK or MCL_1 or BCLXL or BCL2)
BID *= Casp8 or ( Casp2 and not (BCL2 or BCLXL or MCL_1) )
BAK *= (BID and (BIM or BIK or not (BCL2 and BCLXL and MCL_1) ) ) or ( (BIM or BIK) and not (BCLXL or MCL_1) )
BAX *= (BIM and ( (BID or BIK) or not (BCL2 and BCLXL and MCL_1) ) ) or ( (BID or BIK) and not (BCL2 or BCLXL) )
Cyto_C *= BAX or BAK
SMAC *= BAX or BAK
IAPs *= not SMAC or AKT_H
Casp9 *= Casp3 or (not IAPs and Cyto_C)
Casp3 *= (Casp9 and Casp8) or ( Casp3 and (Casp9 or Casp8) ) or ( not IAPs and (Casp9 or Casp8 or Casp3) )
CAD *= Casp3 and Casp9
ATR *= Replication
CHK1 *= ATR
Ca2 *= IP3
DAG *= PLCgamma
DR4_5 *= Trail
ERK *= MEK and not BIK
GF_High *= GF_High
IP3 *= PLCgamma
MEK *= RAF
SOS *= Grb2
Trail *= Trail'''

model_name='CC'

ec_avg_dict={}
manipulation_set=[{'node':'GF_High','enforced_state':1, 'start_time':1, 'end_time':2,'success_probability':1}]
                 #{'node':'GF_High','enforced_state':0, 'start_time':3001, 'end_time':steps,'success_probability':0}]

model = b2p.Model(rules, mode=update_mode)
es=esim.EnsembleSimulation(model,initial_state=initial_state,shuffler=shuffler,ensemble_size=ensemble_size,steps=steps)
es.simulate_manipulated_ensemble(manipulation_set,break_states=break_states)

timeslice_start=0
timeslice_end=steps
df=es.average_state_of_time_slice(timeslice_start,timeslice_end)

df_final_states=pd.DataFrame(es.final_states)
```

```
100%|█████████████████████████████████████████| 100/100 [03:25<00:00,  2.05s/it]
```

In [13]:

```
es.plot_node_evolution_averages(nodes=monitored_nodes, figsize=(10,6), title='PEW enhanced Sizek et al. Cell Cycle model',save_path='figures/Sizek_PEW.svg',linewidth=2)
```

## p\_on -- CAD relationship analysis¶

In [25]:

```
update_scheme_name='async'
update_mode='async'
shuffler=general_async_pick
init_array=np.loadtxt('cell_cycle_init_G0.txt',dtype=str)
initial_state=dict(zip(init_array[:,0],init_array[:,1].astype(bool)))
node_order=list(initial_state.keys())
break_states=[]
ensemble_size=500
steps=10000

CAD_means=[]
CAD_stds=[]
```

In [26]:

```
for p_on in np.linspace(0,1,11):
    print(p_on)
    rules = '''GF *= GF or GF_High
    RTK *= not CAD and (GF_High or GF)
    Grb2 *= RTK and GF_High
    Ras *= Grb2 and SOS
    RAF *= not Casp3 and Ras
    mTORC2 *= PIP3 or not S6K
    PI3K *= Ras or RTK
    PIP3 *= PI3K_H or PI3K
    PDK1 *= PI3K and PIP3
    AKT_B *= not Casp3 and PIP3 and (PDK1 or mTORC2)
    p110_H *= (FoxO3 and not NeddL4) or (p110_H and (FoxO3 or not NeddL4) )
    PI3K_H *= p110_H and RTK and PI3K and Ras
    AKT_H *= AKT_B and p110_H and PI3K_H and Ras and PIP3 and PDK1 and mTORC2
    FoxO3 *= not (AKT_B or AKT_H or ERK) or ( not (AKT_H and (Plk1 or Plk1_H or AKT_B or ERK) ) and not (Plk1 and Plk1_H and ERK) )
    PLCgamma *= RTK and Grb2 and p110_H and PI3K_H and PIP3
    NeddL4 *= Ca2 and IP3
    FoxO1 *= not Plk1 and not AKT_H
    p21_mRNA *= (FoxO1 and FoxO3) or (not Myc and (FoxO1 or FoxO3) )
    TSC2 *= not AKT_H or not (AKT_B or ERK)
    PRAS40 *= not AKT_H and (not mTORC1 or not AKT_B)
    Rheb *= not TSC2 and DAG
    mTORC1 *= not Casp3 and ( (Rheb and not PRAS40) or E2F1 or (CyclinB and Cdk1 and GSK3) )
    S6K *= not Casp3 and mTORC1
    eIF4E *= not Casp3 and mTORC1
    GSK3 *=  not AKT_H and not (S6K and ERK)
    p21 *= p21_mRNA and not Casp3 and not CyclinE
    pRB *= not Casp3 and not CyclinD1 and not CyclinA and (p27Kip1 or not CyclinE)
    p27Kip1 *= not Casp3 and not CyclinD1 and not (Cdk1 and CyclinB) and ( (not CyclinE and (FoxO3 and FoxO1) ) or (not CyclinA and (FoxO3 or FoxO1) ) or (not CyclinE and not CyclinA) )
    Myc *= (ERK and (eIF4E or not GSK3) ) or ( E2F1 and not pRB and (eIF4E or ERK or not GSK3) )
    CyclinD1 *= not CHK1 and ( ( not p21 and ( (not GSK3 and (Myc or E2F1) ) or (Myc and CyclinD1) or (Myc and E2F1) or (E2F1 and CyclinD1) ) ) or ( not pRB and E2F1 and ( (Myc and CyclinD1) or (Myc and not GSK3) or (CyclinD1 and not GSK3) ) ) )
    E2F1 *= not (CAD or CyclinA or pRB) and (E2F1 or Myc)
    CyclinE *= E2F1 and Cdc6 and Pre_RC and not (pRB or p27Kip1 or CHK1 or Casp3)
    ORC *= E2F1 or (Pre_RC and Cdt1 and Cdc6)
    Cdc6 *= not Casp3 and not (f4N_DNA and CyclinA) and ( (E2F1 and ORC and not Plk1) or (Pre_RC and ORC and Cdc6 and Cdt1) )
    Cdt1 *= not geminin and ORC and Cdc6 and not (CyclinE and CyclinA and Cdc25A) and ( (Pre_RC and (E2F1 or Myc) ) or (E2F1 and (Myc or not pRB) ) )
    Pre_RC *= ORC and Cdc6 and Cdt1 and not (Replication and f4N_DNA)
    geminin *= E2F1 and not Cdh1 and not (pAPC and Cdc20)
    CyclinA_mRNA *= not CAD and ((E2F1 and not pRB) or FoxM1 )
    Emi1 *= (E2F1 or not pRB or not p21) and not (Plk1 and CyclinB and Cdk1 and (U_Kinetochores or A_Kinetochores) )
    FoxM1 *= (Myc and CyclinE) or (CyclinA and Cdc25A and Cdc25B) or (Plk1 and CyclinB and Cdk1)
    Cdc25A *= ( (FoxM1 and E2F1 and not pRB) or (not Cdh1 and (FoxM1 or (E2F1 and not pRB) ) ) ) and (not (GSK3 or CHK1) or CyclinE or CyclinA or (CyclinB and Cdk1) )                   
    CyclinA *= CyclinA_mRNA and not pAPC and ( (Cdc25A and (not Cdh1 or Emi1) ) or (CyclinA and ( (not Cdh1 and (Emi1 or not UbcH10) ) or (Emi1 and not UbcH10) ) ) )
    Wee1 *= not Casp3 and not (Cdk1 and CyclinB) and (Replication or CHK1) and (CHK1 or not (Cdk1 and CyclinA and Plk1) )
    UbcH10 *= not Cdh1 or (UbcH10 and (Cdc20 or CyclinA or CyclinB) )
    CyclinB *= (FoxM1 or (FoxO3 and CyclinB)) and not (Cdh1 or (pAPC and Cdc20) )
    Cdc25B *= FoxM1 and f4N_DNA
    Plk1 *= not Cdh1 and (FoxM1 or Plk1_H) and ((CyclinB and Cdk1) or (CyclinA and not Wee1 and Cdc25A) )
    Cdc25C *= f4N_DNA and Plk1 and ((Cdc25B and not CHK1) or (CyclinB and Cdk1) )
    Cdk1 *= CyclinB and Cdc25C and ( not CHK1 or (not Wee1 and Cdk1) )
    pAPC *= (CyclinB and Cdk1 and Plk1) or (CyclinB and Cdk1 and pAPC) or (pAPC and Cdc20)
    Cdc20 *= pAPC and not Emi1 and not Cdh1 and ( not Mad2 or ( not CyclinA and not (CyclinB and Cdk1) ) )
    Cdh1 *= not (CyclinB and Cdk1) and not ( CyclinA and (Emi1 or Cdc25A) )
    Replication *= not CAD and Pre_RC and ( (E2F1 and CyclinE and Cdc25A) or (Replication and CyclinA and Cdc25A and (E2F1 or not f4N_DNA) ) )
    f4N_DNA *= not CAD and ( (Replication and ( (Pre_RC and CyclinA) or f4N_DNA) ) or (f4N_DNA and not Ect2) )
    U_Kinetochores *= f4N_DNA and not Cdh1 and not A_Kinetochores and ( (CyclinB and Cdk1) or U_Kinetochores )
    Mad2 *= U_Kinetochores and not A_Kinetochores
    A_Kinetochores *= f4N_DNA and not Cdh1 and not (pAPC and Cdc20) and ( A_Kinetochores or (U_Kinetochores and Plk1 and CyclinB and Cdk1) )
    Plk1_H *= Plk1 and FoxM1 and (Plk1_H or FoxO3 or FoxO1)
    Ect2 *= f4N_DNA and Plk1_H and Cdh1 and not U_Kinetochores and not A_Kinetochores
    Casp8 *= DR4_5 or Casp3
    Casp2 *= Casp3 or (U_Kinetochores and Mad2 and not (CyclinB and Cdk1) )
    MCL_1 *= (not Casp3 and not Casp2 and ((not GSK3) or (AKT_B and (ERK or not E2F1) ) ) and (not ([%f,0](Cdk1 and CyclinB) and U_Kinetochores) ))
    BCLXL *= (not Casp3 and (BCL2 or not BAD) and ( not U_Kinetochores or ( Plk1 and ( not [%f,0](CyclinB and Cdk1) or (BCL2 and MCL_1) ) ) or  ( (BCL2 and MCL_1) and not [0.5,0] (CyclinB and Cdk1) ) ))
    BCL2 *= (not (Casp3 or BAD or BIM or BIK) and  ( not U_Kinetochores or (MCL_1 and BCLXL) or (Plk1 and (BCLXL or MCL_1 or not [%f,0] (Cdk1 and CyclinB) ) ) ))
    BAD *= Casp3 or not (AKT_H or AKT_B or ERK or S6K) or ( Casp8 and (not (AKT_B and ERK and S6K) and not (AKT_H and (AKT_B or ERK) ) ) )
    BIK *= not (MCL_1 or BCLXL or BCL2)
    BIM *= FoxO3 and GSK3 and not (ERK or MCL_1 or BCLXL or BCL2)
    BID *= Casp8 or ( Casp2 and not (BCL2 or BCLXL or MCL_1) )
    BAK *= (BID and (BIM or BIK or not (BCL2 and BCLXL and MCL_1) ) ) or ( (BIM or BIK) and not (BCLXL or MCL_1) )
    BAX *= (BIM and ( (BID or BIK) or not (BCL2 and BCLXL and MCL_1) ) ) or ( (BID or BIK) and not (BCL2 or BCLXL) )
    Cyto_C *= BAX or BAK
    SMAC *= BAX or BAK
    IAPs *= not SMAC or AKT_H
    Casp9 *= Casp3 or (not IAPs and Cyto_C)
    Casp3 *= (Casp9 and Casp8) or ( Casp3 and (Casp9 or Casp8) ) or ( not IAPs and (Casp9 or Casp8 or Casp3) )
    CAD *= Casp3 and Casp9
    ATR *= Replication
    CHK1 *= ATR
    Ca2 *= IP3
    DAG *= PLCgamma
    DR4_5 *= Trail
    ERK *= MEK and not BIK
    GF_High *= GF_High
    IP3 *= PLCgamma
    MEK *= RAF
    SOS *= Grb2
    Trail *= Trail'''%(p_on,p_on,p_on)

    model_name='CC'

    ec_avg_dict={}
    manipulation_set=[{'node':'GF_High','enforced_state':1, 'start_time':1, 'end_time':2,'success_probability':1}]
                     #{'node':'GF_High','enforced_state':0, 'start_time':3001, 'end_time':steps,'success_probability':0}]

    model = b2p.Model(rules, mode=update_mode)
    es=esim.EnsembleSimulation(model,initial_state=initial_state,shuffler=shuffler,ensemble_size=ensemble_size,steps=steps)
    es.simulate_manipulated_ensemble(manipulation_set,break_states=break_states)

    timeslice_start=0
    timeslice_end=steps
    df=es.average_state_of_time_slice(timeslice_start,timeslice_end)

    df_final_states=pd.DataFrame(es.final_states)


    CAD_means.append(pd.DataFrame(es.final_states).CAD.mean())
    CAD_stds.append(pd.DataFrame(es.final_states).CAD.std())
```

```
  0%|                                                   | 0/500 [00:00<?, ?it/s]
```

```
0.0
```

```
100%|█████████████████████████████████████████| 500/500 [14:59<00:00,  1.80s/it]
  0%|                                                   | 0/500 [00:00<?, ?it/s]
```

```
0.1
```

```
100%|█████████████████████████████████████████| 500/500 [13:47<00:00,  1.66s/it]
  0%|                                                   | 0/500 [00:00<?, ?it/s]
```

```
0.2
```

```
100%|█████████████████████████████████████████| 500/500 [14:07<00:00,  1.70s/it]
  0%|                                                   | 0/500 [00:00<?, ?it/s]
```

```
0.30000000000000004
```

```
100%|█████████████████████████████████████████| 500/500 [15:15<00:00,  1.83s/it]
```

```
0.4
```

```
100%|█████████████████████████████████████████| 500/500 [15:41<00:00,  1.88s/it]
```

```
0.5
```

```
100%|█████████████████████████████████████████| 500/500 [16:02<00:00,  1.92s/it]
```

```
0.6000000000000001
```

```
100%|█████████████████████████████████████████| 500/500 [15:05<00:00,  1.81s/it]
```

```
0.7000000000000001
```

```
100%|█████████████████████████████████████████| 500/500 [15:39<00:00,  1.88s/it]
  0%|                                                   | 0/500 [00:00<?, ?it/s]
```

```
0.8
```

```
100%|█████████████████████████████████████████| 500/500 [15:26<00:00,  1.85s/it]
```

```
0.9
```

```
100%|█████████████████████████████████████████| 500/500 [16:21<00:00,  1.96s/it]
```

```
1.0
```

```
100%|█████████████████████████████████████████| 500/500 [14:22<00:00,  1.73s/it]
```

In [28]:

```
plt.figure(figsize=(8,5))
#plt.title('The change of CAD in function w_on in the [p_on,0] PEW acting on the Cdk1+CyclinB complex\'s links to the antiapoptotic nodes', fontsize=13)
plt.errorbar(np.linspace(0,1,11),CAD_means,yerr=np.array(CAD_stds)/np.sqrt(ensemble_size), linewidth=2)
plt.ylabel('Ratio of CAD=1 in the ensemble', fontsize=16)
plt.xlabel('p_on', fontsize=16)
plt.xticks(fontsize=14)
plt.yticks(fontsize=14)
plt.grid()
plt.savefig('figures/CAD_vs_p_on.svg')
```

In [ ]:

```

```
