# Supplementary material for: Probabilistic edge weights fine-tune Boolean network dynamics
Source: PLoS Comput Biol. 2022 Oct 10;18(10):e1010536. doi: 10.1371/journal.pcbi.1010536 (PMC9584532; doi:10.1371/journal.pcbi.1010536)
Supplement: Attachment — Submitted filename: Responses to reviewers.docx [file pcbi.1010536.s009.docx]

# Response Letter to Reviewers for the manuscript entitled Probabilistic Edge Weights Fine-tune Boolean Network Dynamics (PCOMPBIOL-D-22-00184)

We thank the editorial board of *PLoS Computational Biology* for the management of the review process and giving us an opportunity to respond to the reviewers comments. In this letter we discuss the issues raised by the reviewers and our responses to their points. We highlight the reviewer comments with *italic, Times New Roman* font and our response in regular, Arial font. Modifications of the manuscript are highlighted in blue font.

## Reviewer #1:

We kindly thank the reviewer for the feedback. We found their comments insightful and indeed the suggestions will make for a better paper. We will answer each point in order and reference the modifications made to the manuscript respectively.

*1. On page 3, in the last paragraph, it says that one of the earliest types of noisy BN were BNp in reference [27] which was published in 2013. However, PBN with noise (described in the PBN book from 2010) is much earlier than the BNp paper in reference [27]. Then, on page 4 it says PBNs address the lack of node-specificity of BNps. I think these two paragraphs were presented in a backward order. You could switch the order of the presentation by first describing PBNs as one of the earliest frameworks that introduces noise into the system and then describe that one can use BNps to make the system ergodic as in the case of PBNp.*

We thank the reviewer for this observation indeed it is a valid criticism. Reference [27] is a review paper which also introduces the noisy BN models in the BNp -> PBN order, which we followed. The review paper is indeed published after the PBN reference. However we would argue that the original concept of the perturbed Boolean networks (BNp) is older than the PBN model. We indeed made the error of not referencing the original works. The oldest version we find is from reference a), where the mathematical model is not exactly the same but is very similar to the one described in our paper.

a) O. Golinelli, B. Derrida. Barrier heights in the Kauffman model. Journal de Physique, 1989, 50 (13), pp.1587-1601.

We modified the corresponding text of the manuscript as follows (page 3, last paragraph):

One of the earliest types of noisy BN were the perturbed Boolean Networks (BNp) first suggested in [[27]](https://paperpile.com/c/9sbG9k/4G9w) as Kauffman networks with “thermodynamic noise”. In this paper we use the technical description used in the review article [[28]](https://paperpile.com/c/9sbG9k/gi2nO).

Here reference [27] is the above cited Golinelli et al. paper while reference [28] is the review paper by Trairatphisan et al., originally reference [27].

*2. The framework presented in this paper is very similar to SDDS for which there are methods for parameter estimation (see publication a) below) are well as method for control (see publication b) below). These references could be included when discussing the connections.*

We thank the reviewer for the suggestions, these are indeed highly relevant papers and it was an oversight on our part not to include them.

We modified the text by adding the following paragraphs about parameter estimation and control:

There is an important body of work focusing on parameter estimation and control, which can be readily applied to this framework. In the case of stochastic dynamics in Boolean networks the state space is not necessarily ergodic (though it can have ergodic subsets [a]), however it can be made ergodic by adding a sort of thermodynamic noise (such as in the case of BNp) or a damping factor used in the PageRank algorithm [b], where the system can jump to random states with a nonzero probability. Murrugarra et al. [c] uses the PageRank approach to estimate the stationary probability distribution of states and then applies a genetic algorithm to estimate the node propensity parameters (akin to the PEW tuples) of the SDDS model, given a desired final probability distribution on the state space, such as more balanced attractor basins. We argue that this method can be expanded to our PEW model in a straightforward way, to estimate edge-weights.

Controlling stochastic dynamic systems is a challenging task. Aguilar et al. proposes a near optimal method in [d] for estimating control policies in stochastic Boolean networks, which similarly to the parameter estimation method by Murragarra et al. [c], can theoretically be expanded to the PEW framework. In both the parameter estimation and the control methods the state-space of PEW Boolean models have to be made ergodic. One way of doing that is the one proposed in Appendix 1 where we reproduce the BNp variant with a universal noise.

Investigating the different methods of stable motif control [e,f] combined with PEW Boolean models could be a relevant next step. We show that noisy edges in stable motifs have a significant impact on the emergent dynamics, due to the fact that stable motifs are crucial in locking in attractor states.

[a] Ribeiro, A. S., & Kauffman, S. A. (2007). Noisy attractors and ergodic sets in models of gene regulatory networks. *Journal of theoretical biology*, *247*(4), 743-755.

[b] Page, L., Brin, S., Motwani, R., & Winograd, T. (1999). *The PageRank citation ranking: Bringing order to the web*. Stanford InfoLab.

[c] Murrugarra, D., Miller, J., & Mueller, A. N. (2016). Estimating propensity parameters using Google PageRank and genetic algorithms. *Frontiers in Neuroscience*, 513.

[d] Aguilar, B., Fang, P., Laubenbacher, R., & Murrugarra, D. (2020). A Near-Optimal Control Method for Stochastic Boolean Networks. *Letters in biomathematics*, *7*(1), 67.

[e] Zanudo, J. G., & Albert, R. (2015). Cell fate reprogramming by control of intracellular network dynamics. *PLoS computational biology*, *11*(4), e1004193.

[f] Rozum, J. C., Gómez Tejeda Zañudo, J., Gan, X., Deritei, D., & Albert, R. (2021). Parity and time reversal elucidate both decision-making in empirical models and attractor scaling in critical Boolean networks. *Science Advances*, *7*(29), eabf8124.

*3. The initial states were not specified in Figure 2. Were these selected at random?*

We thank the reviewer for noticing this missing information, Indeed the initial state is only implied on Figure 2, it being the epithelial attractor from which we induce the EMT by overexpressing TGFB. We added the following sentence to the paragraph discussing the figure:

The initial state of the simulated networks is the epithelial attractor (Ecaderin=miR200=ON; all other nodes OFF).

*4. What does it mean that the stable motifs don’t loose their nonlinear attributes? What nonlinear attributes are you referring to in the caption of Figure 3?*

We agree that this should have been clarified. The statement refers to the fact that although we add a slight noise to the stable motif consisting of ZEB and miR200 in the “wild type” case (Figure 3 left panel) the transition exhibits the bistable-like transition, as compared to the much more noisy mutant case (right panel). We imply this in the paragraph:

“Figures 2 and 3 show two additional model results that recapitulate the experimental data: first, the MET is significantly quicker in the mutant system compared to the wild type, and second, the ***mutant version loses its bistability, shown by the red median line of the E-cadherin distribution averaged in the interval of timesteps between 100 and 150.*”**

To make it more explicit we added the following sentence to this paragraph:

However, the small amount of added noise in the “wild type” version on the edge ZEB - miR200 edge preserves the bistable transition with the increased TFGB concentration (Figure 3 left panel) we expect with a noiseless stable motif, while still allowing the MET to happen due to the fact that the feedback loop is not irreversibly locked in.

*5. Explain the axes of Figure 3. What is depicted in the horizontal axis?*

We thank the reviewer for the observation. We added ax labels to the figure both in the manuscript and in the Jupyter notebook. The horizontal axis represents the ensemble average values of E-cadherin within the time-interval specified (steps 100 to 150).

*6. Figure 4 appears on two separate pages. Merge the two panels so that they show up on the same page.*

The panels are now merged.

*Minor Revisions:*

*1. On page 3, at the end of the second paragraph, it says “truly random”. It would be is best to remove the adjective “truly” and just go with random as it might create confusion.*

We agree with the reviewer, the word “truly” is now removed.

*2. It seems the acronym for the Transforming Growth Factor-beta should be TGF-beta instead of TGFB.*

This is indeed the more accurate acronym, however we have met instances in the literature where the character “beta” is simply substituted for the latin B. We just find this acronym more convenient but we would kindly ask the editor to decide, which version do they prefer in PLoS and we would adapt to that.

## Reviewer #2

First of all we thank the reviewer for their feedback, it indeed helped us improve and better understand the finer details of our method. We think that there might be a misunderstanding of what we propose in this paper. We do not propose a better version of the Boolean modeling framework, but we offer a tool where the relative strength of individual edges can play an important role in the dynamics. This alteration in relative strength can be achieved by adding some sort of noise (specified by a noise function) dependent on the state of the source and/or target node states. We implement this tool as a modification of the popular BooleanNet software and show a few examples where adding a few PEW operators can significantly improve the biological plausibility of Boolean models, thus increasing their predictive capability. But it is indeed not intended as a replacement for any well established methodology by any means.

*1) The presentation of the probabilistic grammar is clear, but the type of modeling output(s) is not described. Is it time-dependent? Probabilistic over Boolean node states? Probabilistic over the full set of Boolean node states?*

The parameters and the noise function of the PEW operators are *not* time-dependent. It is technically possible to make them time-dependent (by changing the parsed rules during a simulation) but we don't do this in any of our applications. A PEW operator applied in the rule of a single node will potentially make the *target node* stochastic. This also means that all nodes *downstream* of the stochastic node can behave stochastically as a result.

In the applications we use the ensemble average evolution of nodes to represent the average emergent effect of the stochasticity added to the system. For example if a node has a 50% noisy response to constant ON stimulation, in that case the ensemble average will settle to a “concentration” of 0.5 when its input is on. This of course, combined with other noise operators and the complex nature of regulatory networks can have non-trivial emergent effects, as we can see in the applications.

We added the following clarification to the main manuscript:

The parameters and the noise function of the PEW operators are *not* time-dependent. Nonetheless, it is technically possible to make them time-dependent (by changing the parsed rules during a simulation) but we don't do this in any of our applications. A PEW operator applied in the rule of a single node will potentially make the *target node* stochastic. This also means that all nodes downstream of the stochastic node can behave stochastically as a result. The combination of noise operators with the complex nature of regulatory networks can have non-trivial emergent effects, as we see in the applications.

*2) What is the mathematical framework of this approach? Is it a discrete-time Markov chain? It seems to me that it is a special case of Probabilistic Boolean Networks, because there are different transition rules for a node, weighted by probabilities. Can the authors demonstrate that their approach is (or is not) a special case of Probabilistic Boolean Networks?*

The reviewer raises some important questions in this point and by answering them we learned a lot more about our own model. Generally, as operators are defined by a noise function and a set of parameters, it depends on the noise function what the exact mathematical framework is. Nonetheless adding noise to a single edge can make the target node stochastic. Stochastic nodes indeed “transform” the state transition graph (STG) of the Boolean model into a Markov chain. However, just as in the case of Probabilistic Boolean Networks, the Markov chain is not necessarily ergodic but it can have ergodic subsets, due to the fact that only a subset of nodes is stochastic, while other non-stochastic parts of the network can still lock into states permanently. For the purposes of our paper we chose the representation/analysis of the dynamic evolution of models as ensemble averages of states (started from specific, biologically relevant initial conditions), instead of a more general STG/Markov chain analysis (e.g. determining the stationary probability of states within ergodic subsets, PageRank, etc. ). We do this mainly because the ensemble avarages capture relevant time evolution patterns and the stochasticity in the case of certain nodes emerges as a nonbinary average in the steady state, akin to intermediate concentrations of molecules in cells.

Regarding the relationship to PBNs we found that indeed, in the case where the noise function is a Bernoulli coin toss (draw from a binomial distribution), PEW models can be transformed into PBNs. We also show that using the same logic PBN function sets can be transformed into probabilistic edge weights. The details of this analysis are discussed in a separate supplementary document attached to this response.

We added the following modifications to the main manuscript: we modified the paragraph comparing our model to PBNs and referenced the Supplementary Document 1 describing their relationship. Moreover, we added the following paragraph to the theoretical description:

The exact mathematical framework of the PEW approach depends on the noise function applied in the operators, nonetheless adding any kind of noise to a single edge can make the target node stochastic. Stochastic nodes indeed transform the state transition graph (STG) of the Boolean model into a discrete Markov chain. However, just as in the case of Probabilistic Boolean Networks, the Markov chain is not necessarily ergodic but it can have ergodic subsets, due to the fact that only a subset of nodes is stochastic and other non-stochastic parts of the network can still lock into states permanently. For the purposes of our paper we chose the representation/analysis of the dynamic evolution of models as ensemble averages of states (started from specific, biologically relevant initial conditions), instead of a more general STG/Markov chain analysis (e.g. determining the stationary probability of states within ergodic subsets, PageRank, etc. ). We do this mainly because the ensemble averages capture relevant time evolution patterns and the stochasticity in the case of certain nodes emerges as a nonbinary average in the steady state, akin to intermediate concentrations of molecules in cells.

*3) Although the authors show applications where their approach is efficient (in the first example) and more elegant (in the second), I am not convinced that it is a major improvement of actual Boolean approaches. For that, the authors should show that a lot of biological reactions/influences are impossible to implement in any actual Boolean approaches and are only possible within their approach.*

The following argument is true for both applications however in the second application is probably a bit more illustrative. The reason we chose the model of the second example (Sizek et al. model) is that in this case it is critical to *silence edges instead of nodes*. Typically in in-silico simulation of Boolean models we probabilistically silence the activity of nodes (see Sizek et. al 2019) when we want to mitigate a certain effect caused by their activity. In this case this effect is the downregulation of the anti-apoptotic nodes caused by the activation of CyclinB/Cdk1. However, the CyclinB/Cdk1 complex’s undisturbed progression is critical for a healthy cell cycle progression, thus silencing the nodes themselves would cause more harm than good (disturbing the healthy cell cycle progression). However silencing the edges inhibiting the anti-apoptotic nodes to a certain degree (without just removing them) mitigates the unwanted effect significantly. It is theoretically possible to implement the same effect by using PBNs, yet we would argue that in this case modifying the edge-weights may be more intuitive.

Moreover the general case of the PEW operators allows us to use different functions, other than the draw from a binomial distribution (which we use in the case of the two main applications and is the default function). The PBN equivalence is only true for this latter case. Alongside the manuscript we offer a more general Python implementation, where the alternative function can also be named in the operator, and even PEW operators using different functions can be mixed in the same Boolean model. The new syntax is [f,w1,w2], and not specifying a function makes the operator default to the binomial draw. Any number of noise functions can be defined in a separate file. To demonstrate the versatility of our model we added another supplementary document, an Application Note, with a few more, simpler applications.

We modified the manuscript by explaining the use of PEW operators with general noise functions and we added references to the Application Note.

Using the [w_on, w_off] syntax, the code defaults to the Bernoulli coin-toss as the noise function. Yet generally one can also specify the noise function in the operator: **[f_name, p1, p2]**, where the “f_name” is a function defined in a separate Python file (PEW_functions.py) and p1, p2 are parameters of the function. The parser interprets each operator as a separate entity, so operators with different noise functions can be mixed in the same model. To see use cases of this general formula please see the Application Note (Supplementary Document 2) and its accompanying Jupyter notebook.

With all these responses and modifications in place we hope our manuscript will be fit for publishing in PLoS computational Biology.
